# Enhanced security for medical images using a new 5D hyper chaotic map and deep learning based segmentation

**DOI:** 10.1038/s41598-025-04906-4

**Published:** 2025-07-02

**Authors:** S. Subathra, V. Thanikaiselvan

**Affiliations:** School of Electronics Engineering, Vellore Institute of Technology, Vellore, Tamil Nadu India 632014

**Keywords:** Hyper-chaotic map, Image encryption, Dynamic DNA encoding, Critical region segmentation, U-Net, Zig-zag scrambling, Computer science, Information technology, Electrical and electronic engineering

## Abstract

Medical image encryption is important for maintaining the confidentiality of sensitive medical data and protecting patient privacy. Contemporary healthcare systems store significant patient data in text and graphic form. This research proposes a New 5D hyperchaotic system combined with a customised U-Net architecture. Chaotic maps have become an increasingly popular method for encryption because of their remarkable characteristics, including statistical randomness and sensitivity to initial conditions. The significant region is segmented from the medical images using the U-Net network, and its statistics are utilised as initial conditions to generate the new random sequence. Initially, zig-zag scrambling confuses the pixel position of a medical image and applies further permutation with a new 5D hyperchaotic sequence. Two stages of diffusion are used, such as dynamic DNA flip and dynamic DNA XOR, to enhance the encryption algorithm’s security against various attacks. The randomness of the New 5D hyperchaotic system is verified using the NIST SP800-22 statistical test, calculating the Lyapunov exponent and plotting the attractor diagram of the chaotic sequence. The algorithm validates with statistical measures such as PSNR, MSE, NPCR, UACI, entropy, and Chi-square values. Evaluation is performed for test images yields average horizontal, vertical, and diagonal correlation coefficients of –0.0018, –0.0002, and 0.0007, respectively, Shannon entropy of 7.9971, Kolmogorov Entropy value of 2.9469, NPCR of 99.61%, UACI of 33.49%, Chi-square “PASS” at both the 5% (293.2478) and 1% (310.4574) significance levels, key space is 2^500^ and an average encryption time of approximately 2.93 s per 256 × 256 image on a standard desktop CPU. The performance comparisons use various encryption methods and demonstrate that the proposed method ensures secure reliability against various challenges.

## Introduction

In recent decades, a trend has been toward implementing revolutionary healthcare infrastructure, including digital health services, remote surgical assistance, image-based diagnosis, real-time health monitoring devices, and digitising patient medical data. The development of information exchange and the expansion of networks have led to security concerns in medical image information. The imaging modalities extract internal image features. The security of medical imaging has been increasing in importance in recent years. Medical images should be transmitted securely, ensuring privacy, integrity, and dependability. Patient data is highly valuable, encompassing personal health information and financial details, making healthcare data particularly sensitive and critical. Privacy and security issues in the digital health era are complex and multifactorial.

By considering enormous collections of medical imaging and patient record data, automated illness analysis is made feasible, greatly enhancing the precision and effectiveness of medical diagnostics. When medical data is managed and shared with external parties, as in publicly accessible, privately stored, or mixed cloud environments, many problems regarding its confidentiality and integrity may arise. However, the healthcare industry has ongoing security risks to medical data. A minor change in the medical images results in a life-threatening situation for the patients. Therefore, a reliable protection solution is required to transfer confidential medical information securely. Encrypting images, among other protection techniques, is a ubiquitous and efficient way to safeguard image data. Image encryption increases resilience against security attacks by using a mathematical method to transform the original image into a format that is difficult to interpret. Over the past few decades, researchers have presented numerous image encryption schemes employing various methodologies. The high redundancy and massive pixel density of medical images make traditional encryption techniques ineffective. Conventional encryption techniques, which depend on intricate mathematical operations, require much processing power. They are also resource-intensive and lack scalability, especially when working with extensive multimedia datasets or carrying out batch operations. They are also vulnerable to interference and attacks, such as custom-plaintext and data analysis attacks^1^**.**

Chaotic system image encryption has been used by scientists and professionals to address these problems. The use of chaos in cryptography has gained popularity in recent decades because of its essential characteristic of being hypersensitive to initial conditions, which produces chaotic sequences that appear random. The permutation and the diffusion are the two stages that usually comprise the chaos system-driven picture encryption framework^2^**.** The pixel rearrangement method, known as permutation, has altered the image into an unrecognisable format, which shifts the pixel positions throughout the image while maintaining the same pixel values. Since the first phase is inadequate and attackers can readily exploit it, the system implements the diffusion or spreading phase. As a result, chaotic systems generate a pattern that sequentially modifies the pixel values of the entire image when executing the spreading phase with a chaotic map. The system repeats the scrambling-spreading procedure multiple times until it attains sufficient security.

One of the most efficient ways to protect medical image information is through cryptography^3^**.** However, the information becomes vulnerable to attackers when it is decoded. Transferring and securing the key is very important for the authorised user of the algorithm. The key size increases the computational cost. Safety and confidentiality are affected when an unauthorised person discloses the key. Even the authorised individual may find it challenging to access the encrypted data in critical situations. A sophisticated encryption approach that considers these issues is necessary to provide total data safety.

Intelligent encryption using deep learning is gaining widespread popularity as a medical image encryption tool in digital health platforms. Integrating deep neural network architecture with chaotic systems holds significant potential for improving cryptographic techniques. Deep learning-powered picture encryption methods meet modern multimedia content security requirements. Several deep learning-based image encryption methods have been developed to protect privacy while computation is on. Artificial intelligence (AI) can produce complicated and resilient encryption algorithms that are challenging for unauthorised parties to crack. However, the amount and accuracy of training datasets determine how well AI secures medical images by increasing computational complexity^4^**.** Prognostic modelling, therapeutic decision-making, personalised treatment, and image interpretation can leverage deep learning strategies for better outcomes. In contrast to other deep learning frameworks, CNN-driven architectures have become increasingly popular for encrypting medical images. This work addresses the current challenges of medical image encryption by developing innovative image encryption that combines deep learning models, such as the U-Net network, with a novel 5D hyperchaotic system. The key generation uses U-Net for segmenting the significant features in medical images, for example, anatomical structures, tumour cells, and vessels/veins. The novel 5D hyperchaotic system generates unique and large random sequences using the initial conditions of segmented key values of the medical images. The novel hyperchaotic sequence and dynamic DNA encoding perform different stages of the encryption process in medical images, improving the security and privacy of individual health records. A two-stage permutation and diffusion process are used, along with a hyperchaotic system and dynamic DNA encoding. Dynamic DNA encoding with a hyperchaotic system gives significant parallelism, increased data density, and extremely low power utilisation. For the medical segmentation of complex forms, U-Net’s unique structure, which includes an encoder and a decoder, allows the network to preserve spatial detail while collecting contextual information. The proposed work integrating the hyperchaotic system with U-Net architecture provides efficient encrypted images and authenticated health data transfer.

Impact of deep segmentation network on the encryption technique:

- 1. U-Net removes critical regions of the medical image, and it is used for key generation.
- 2. The statistical data of critical regions can be used as dynamic encryption keys, so each image has a unique set of initial conditions.
- 3. The protection of medical images is increased with different sets of keys in the encryption algorithm.
- 4. U-Net can maintain high segmentation accuracy while reducing computing overhead because it can function well on small datasets with few training data.
- 5. The U-Net architecture preserves spatial information and ensures precise extraction of important image features by combining skip connections with an encoder-decoder design.
- 6. Segmenting critical regions is a single preprocessing step for each image with the lowest computing cost.
- 7. Training time and resource consumption are reduced when U-Net is trained on a small, specifically chosen dataset.
- 8. Stable key generation is guaranteed by constant and dependable segmentation across various images.

The proposed work is validated by calculating the statistical parameters and performing security analysis. The simulation results shows that the proposed work is validated for medical image encryption.

The key contributions of this proposed work are as follows:

- A New 5D Hyperchaotic map is proposed for secure encryption.
- Critical region-based key generation utilising U-Net segmentation to produce unique initial conditions for a novel 5D hyperchaotic map. Although the computational complexity has been marginally elevated due to U-Net, the key generation remains highly robust against security attacks.
- An encryption strategy combines zig-zag scrambling, dynamic DNA encoding, Dynamic DNA flipping and XOR-based diffusion guided by chaotic sequences.
- Comprehensive security analysis demonstrates robust resistance against common security attacks, ensuring the system’s reliability and protection. Quantitative performance evaluation on medical images, of the proposed method, achieves horizontal, vertical, and diagonal correlation coefficients of ≤|0.002|, Shannon entropy ≥ 7.9971, NPCR ≥ 99.61%, UACI ≥ 33.49%, Chi-square “PASS” at both 5% and 1% levels, large key space of 2^500^ and encrypts a 256 × 256 image in ≈ 0.018 s demonstrating both high security and computational efficiency of the algorithm.

This article presents the following sections: Section “Literature survey” discusses detailed literature surveys and contributions. Section “Preliminaries” outlines the preliminaries of the novel 5D hyperchaotic map and customised U-Net. Section “Proposed block diagram” explains the proposed block diagram and algorithm. Section “Experimental results” presents the experimental results. Section “Performance analysis” provides performance analysis, and Section “Performance comparison with existing algorithms” demonstrates a Performance Comparison with Existing Algorithms analysis of Computational complexity and Time Performance. Finally, Sect. “Conclusion and future scope” concludes the work and gives future directions.

## Literature survey

### Medical image encryption

The survey on medical image encryption demonstrates a different technique targeted at enhancing security and retaining efficiency. The paper^5^ proposes a new 1D-Cosine within Sine (1D-CwS) to permute and diffuse the medical images. The new chaotic map generates expanded random numbers, which are validated using the Lyapunov parameters and bifurcation plot. It resists the various attacks generated while transferring the medical images. The research^6^ presents Elliptic Curve Cryptography (ECC) and the Blum-Goldwasser Cryptosystem (BGC) to improve the encryption techniques in medical images. The algorithm’s adaptability to cipher keys increases the potential of the system to be resilient to threats. The method reduces the computation time, increases the entropy, and attains critical values in NPCR. Recently, the idea of DNA encoding in conjunction with chaotic sequences has gained popularity among academics studying permutation and Diffusion. The proposed technique^7^, a novel 2D Cosine-Sine map, generates the chaotic sequence for medical video confusion, and Diffusion uses chaotic sequences with dynamic DNA. The result proved that the technique based on chaotic sequences with dynamic DNA improves the security of medical videos. Researchers have also advanced the encryption technique for confidential areas^8^in medical images using Rubik’s cube-based bit plane shuffling with a logistic map. It reduces the algorithm’s complexity by considering the confidential area of medical images. Advantages include high security against data loss. The dual-image encryption technique^9^ was proposed in this paper by combining chaotic phase Fresnel diffraction with a fingerprint key. It guarantees enhanced security with strong robustness against noise attacks, plain test attacks, occlusion attacks, ciphertext attacks, and histogram attacks.

Another methodology proposed by the paper^10^ uses a biometric image as the key generated by extracting texture features in the circular pattern and finding spatial weighted averaging for the key image. The one-dimensional fractional trigonometric function chaotic map performs permutation and diffusion between the original image and the biometric fused key image. This method demonstrates strong sensitivity, robustness, and resistance to attacks. The technique proposed by^11^ utilises two logistic maps initiated by a four-power bit grey-level code. The algorithm encrypts the COVID-19 X-ray images with different sizes. Due to the more extended key size, the encrypted image is infeasible for a forced attack. The medical image encryption is enhanced in this paper^12^ by combining Chen’s chaotic map, Henon’s chaotic map, and Brownian motion. The technique attains the expected security level by analysing different statistical parameters such as pixel correlation, homogeneity, histogram, entropy, NPCR, and UACI analysis. Another new approach investigated in this paper^13^ is the integer wavelet transform combined with DNA and a chaotic map to obtain secure medical images in the transform domain. The technique obtains maximum entropy and outperforms the previous methods by applying two-stage shuffling and diffusion processes.

The paper^14^ introduced a new reconfigurable non-volatile array-type memristor implemented using a CMOS circuit to reduce the manufacturing cost, and it is used for power load forecasting. The existing work discusses deploying a chaotic system for data security in the memristor-based power load prediction. The complex dynamic behaviours are obtained by memristive FN-HNN neural networks, which can be used for image encryption applications to improve information security performance^15^. In this work^16^, the memristive array is introduced for parallel neural network computations to update the LSTM weights in the fault diagnosis operation. They achieved faster convergence and 98% accuracy, and this parallelisation strategy generalises to derive multiple secret keystream subblocks per cycle for image encryption. The research work^17^ proposed a memristive memory circuit with association capability to mimic the bioinspired brain network. The three-module memristive circuit emulates Drosophila associative learning (synapse/neuron, cross-modal synergy, and threshold detection) is implemented. While aimed at bionic AI, its adaptive weight modulation suggests a hardware approach for context-aware key updates, equivalent to adaptive diffusion rounds keyed by stimulus intensity in image encryption.

In this paper^18^**,** the source image is divided into a subblock of 8 × 8, and the transformation table is generated from a novel two-dimensional enhanced coupling Quadratic map and a dimension Tent Logistics map with a hash function. The scrambling is performed based on the Tent Logistics map, and the diffusion is implemented with the help of the transformation table, reducing the encryption method’s complexity. The paper referenced in^19^ gives asymmetric key management using an Elliptic Curve Diffie-Hellman (ECDH), Elliptic Curve Cryptography (ECC), and SHA-256 functions. DNA encoding integrates with the novel two-dimensional cross-sine-modular model(2D-CSMM) to construct the enhanced S-box to implement global shuffling and chaotic rotation to obtain the efficient cipher image.

The literature survey found that lightweight medical picture encryption systems with excellent transmission efficiency are necessary for real-world medical applications. However, medical image security and privacy must also be guaranteed to prevent unauthorised individuals from accessing them. The approaches used in existing methods are susceptible to complex attacks, have high computational complexity, and are limited in their capacity to handle big data sets. These issues show how difficult it is to achieve broader usability in real-time health information.

### Medical hyperchaotic image encryption

Chaos-based image encryption algorithms have gained popularity across industries, but many current methods rely on low-dimensional chaos, which may compromise security. This study presents a 6D high-dimensional chaotic system combined with DNA encoding^20^ to enhance encryption robustness. The process involves DNA-level diffusion and shuffling, rearranging the original image sequences using random chaotic sequences. Experimental results show that the algorithm increases image complexity, reduces pixel correlation, improves entropy (near 8), and offers resilience against geometric and cut-off attacks. Another study proposes a 5D hyperchaotic system^21^ with quadratic nonlinearity for image encryption, using Hybrid Random Matrix Transform (HRMT) for confusion and diffusion processes. The investigation demonstrates the algorithm’s performance and durability against attacks.

In reference^22^**,** the encryption scheme generates a cipher image by combining Fisher-Yates confusion, Chen chaotic system, and DNA operation. After scrambling, DNA addition, XOR operation, and subtraction are performed between DNA sequences to increase the algorithm’s accuracy. Another novel approach is introduced in this paper^23^**,** which uses a random signal insertion as an initial condition for a 6D chaotic map. Each 8-bit pixel is divided into two 4-bit pixel parts and creates a new pixel matrix. Confusion, circular shift, and diffusion are performed in the new pixel matrix, improving the encryption scheme’s effectiveness. It also increases the key space and enables it to endure various attacks. The paper^24^ proposes an algorithm that combines integer wavelet transform, a new 3D hyperchaotic map, a bit-level permutation, and random DNA shuffling using a DNA base cube to guarantee the security of medical images. The study analysis in^25^ executed a 4D large-scale hyperchaotic map (4D-LSHM) for cross-permutation and shuffling in the different bit-level slices of the image. Compressed sensing is implemented along with encryption to improve the performance algorithm by reducing the size of the cipher image. Thus, the literature analysis shows that using hyper-chaotic maps such as 6D and 5D with DNA encoding reduces pixel correlation and substantially improves security. However, many encryption techniques depend mainly on chaotic maps for confusion and diffusion, limiting the algorithm’s flexibility if the chaotic map is compromised. So, real-time application issues demand further advances in widespread deployment.

### Preliminaries

#### New 5D hyper chaotic system

**Fig. 1:**
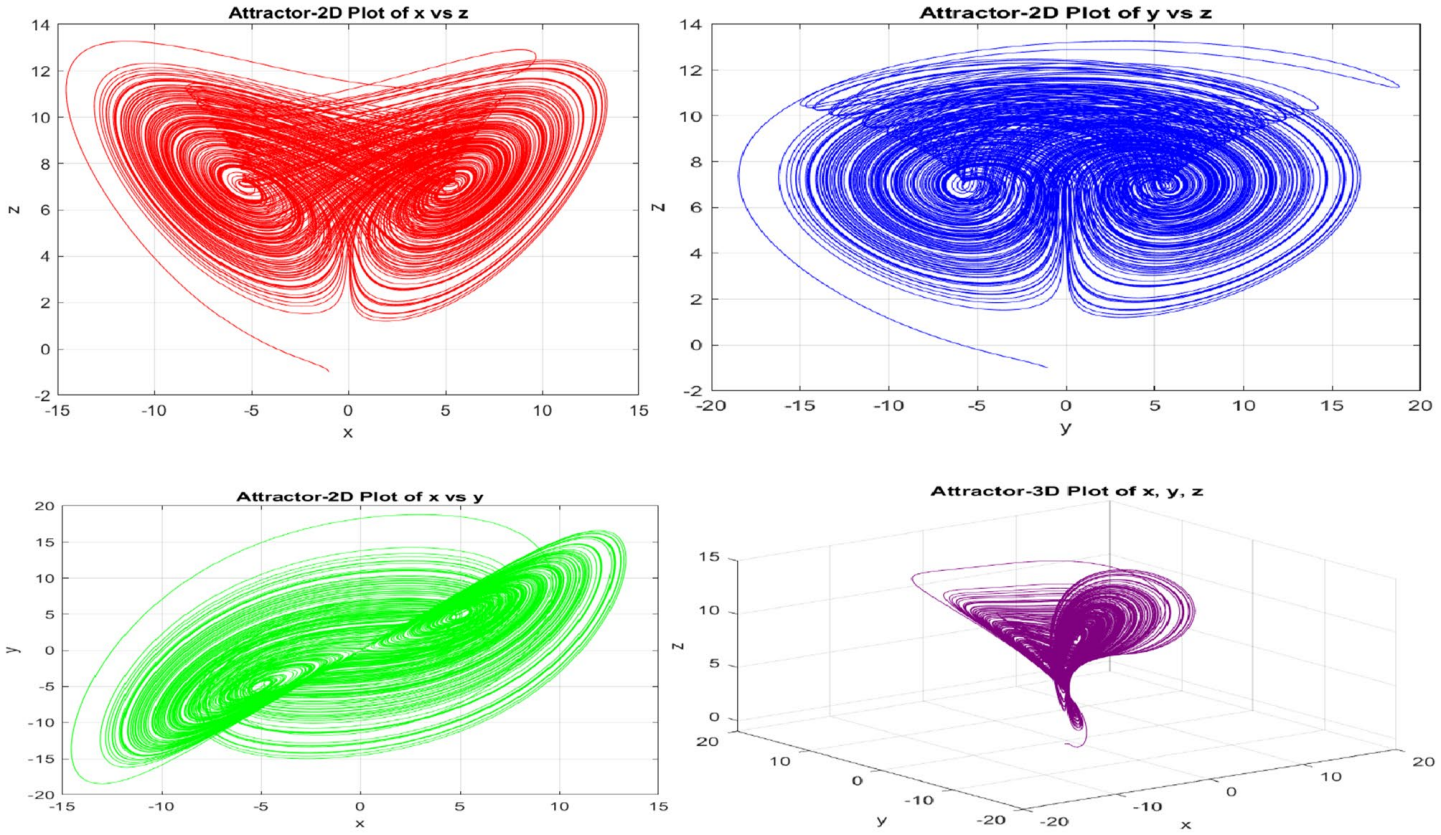
Attractor diagram for NEW 5D Hyper chaotic system.

**Fig. 2:**
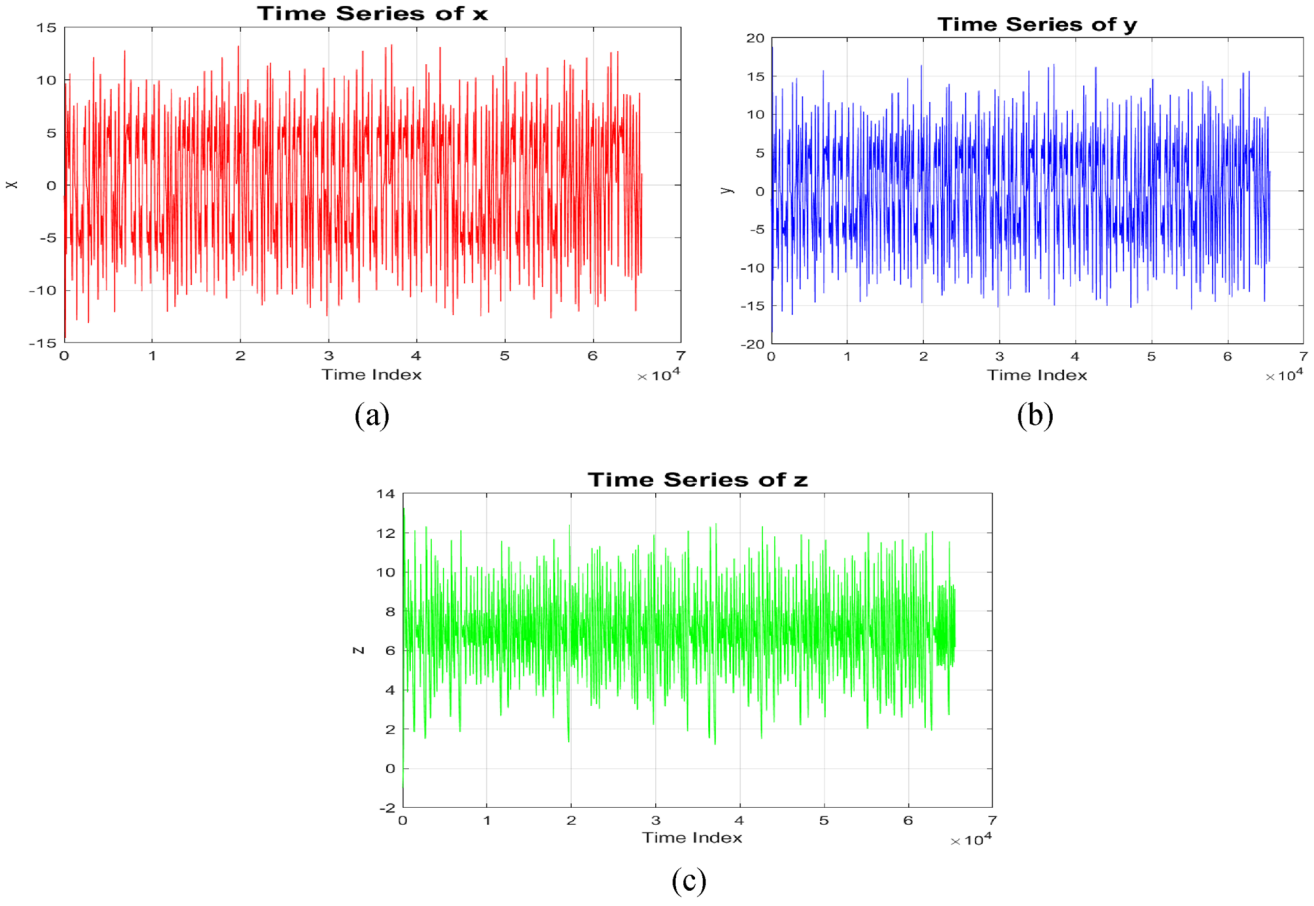
2D-time series plot: (**a**) 2D-time series plot for chaotic sequence-X, (**b**) 2D-time series plot for chaotic sequence-Y (**c**) 2D-time series plot for chaotic sequence-Z.

**Fig. 3:**
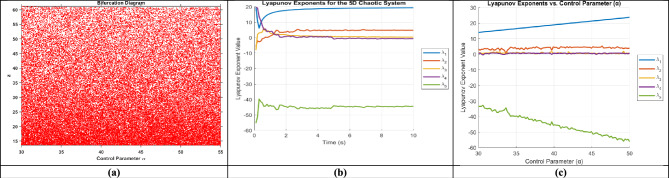
(**a**) Bifurcation diagram of New 5D Hyperchaotic system, (**b**) Lyapunov exponent of 5D chaotic system concerning time (**c**) Lyapunov exponent of 5D chaotic system to the control parameter α.

In the preliminary section of this article, we introduce a novel 5D hyperchaotic system that generates a chaotic sequence of five variables (x, y, z, u, and v), evolving dynamically over time. This dynamic, chaotic sequence ensures a non-linear and secure encryption process. The higher-dimensional phase space of the 5D chaotic system allows for greater complexity in trajectories and increases the system’s unpredictability, which makes it advantageous for encryption. This additional intricacy intensifies the diffusion and confusion effects essential for safe picture encryption. The control parameter values are α = 40, β = 8, $\gamma$ ** =** 40, ∂ = 1, €= −0.5, ϑ = −0.5, ρ = 25.5 and κ = 0.05. The statistics of critical regions of the medical image extracted from the U-Net network provide the initial conditions for the novel 5D chaotic map. Fig. 1 gives the attractor diagram, and Fig. 2 displays the time series plot of the New 5D hyperchaotic sequences. Fig. 3(a) represents the bifurcation diagram of the new 5D hyperchaotic system, and the bifurcation diagram represents complex, chaotic dynamics by exposing densely populated plot regions. The equations governing the New 5D hyperchaotic system are given in Eq. (1)

1 $$\left.\begin{array}{c}\frac{dx}{dt} = \gamma \left(y-x\right)+\kappa y+x \\ \\ \frac{dy}{dt} = \gamma x+\partial y-x{z}^{2}+yz \\ \\ \frac{dz}{dt} = -\beta z+{x}^{2}+xy+\kappa z\\ \\ \frac{du}{dt} = \text{\euro}y+ \vartheta u\alpha \\ \\ \frac{ dv}{dt} = \rho x+\kappa v+z \\ \end{array}\right\}$$

The Lyapunov exponents (LEs) characterise the chaotic nature and dimensionality of the attractor. The LEs are computed using the variational equations derived from the Jacobian matrix of the system, given in the Eq. (2)

2 $$\left[\begin{array}{ccccc}-\gamma + 1& \gamma + \kappa & 0& 0& 0\\ \gamma - {z}^{2}& \partial + z& -2xz +y& 0& 0\\ 2x+y & x& \beta + \kappa & 0& 0\\ 0& \text{\euro}& 0& \vartheta \alpha & 0\\ \rho & 0& 1& 0& \kappa \end{array}\right]$$

The Lyapunov exponents are calculated using the QR decomposition method over many iterations, where QR refers to the decomposition of a matrix into an orthogonal matrix Q and an upper triangular matrix R. The time evolution of LEs is obtained from Eq. (3)

3 $${\lambda }_{\begin{array}{c}i \\ \end{array}}= \underset{t \to \infty }{\mathit{lim}}\frac{1 }{t} \sum_{k}^{t}\mathit{log}\left[\begin{array}{c}{R}_{i i} (K)\end{array}\right]$$

where ${{\varvec{R}}}_{{\varvec{i}}{\varvec{i}}\boldsymbol{ }}\left({\varvec{K}}\right)$ represents the diagonal elements of the R matrix obtained from QR decomposition at iteration K. The calculated Lyapunov exponents for the hyperchaotic system are λ_1_ = 19.3889, λ_2_ = 4.7545, λ_3_ = 0.3866, λ_4_ = −0.5982, and λ_5_ = −44.4191. The positive Lyapunov exponent represents the hyperchaotic nature of the system. The time histories of the Lyapunov exponent (λ) and control parameter (α) are shown in Fig. 3(b) and 3(c), respectively.

##### Kolmogorov entropy of the new 5D hyperchaotic system

**Fig. 4:**
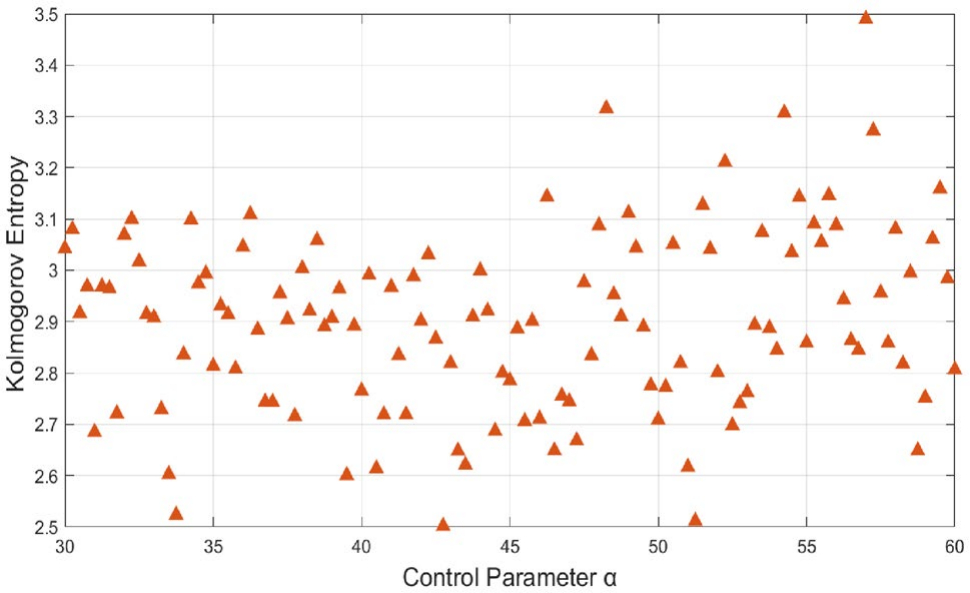
(**a**) Kolmogorov entropy of new 5D Hyperchaotic system.

The Kolmogorov entropy (KE) calculates a system’s predictability, called the Kolmogorov–Sinai entropy. As long as two distinct locations on the attractor are spaced at a scale smaller than a threshold length, Kolmogorov entropy can be calculated by tracking them about time. Due to initial condition sensitivity, it assesses a system’s unpredictability by measuring how rapidly state-related information is lost. Finding Random points along a trajectory in phase space is an effective method for measuring KE and the reference paper^26^ details of KE calculation. Kolmogorov entropy is arbitrarily high for a stochastic system and equals zero for a predictable or fully periodic system. Thus, the KE entropy can estimate the nonlinear system’s chaos and complexity of motion. Table 1 compares KE with existing methods, and the high value of KE for our method ensures that it generates unpredictable data at the fastest rate. Figure 4**.** plots the KE values of the proposed method; it shows most of the values lie above 2.5, indicating the high chaotic property of the proposed system.

**Table 1:** Comparsion analysis of kolmogorov entropy.

Reference	Proposed system	Kolmogorov entropy
REF^27^.	Hyperchaotic 2D sinusoidal exponential memristive system	1.6428
REF^28^.	4D Non-degenerate chaotic system (4D-NDCS)	1.94
REF^29^.	2D Salomon map	2.4624
REF^30^.	2D Zettle map	2.5026
REF^31^.	2D Price map	2.4530
**Proposed**	**New 5D hyperchaotic system**	**2.9469**

### NIST test

NIST SP 800–22 Statistical Test Suite^32^ is a well-known framework designed to assess the statistical randomness of binary sequences produced by chaotic systems or pseudorandom number generators. It is necessary for validating the security and unpredictable nature of sequences employed in communication networks, encryption, and other applications where randomness is crucial. The sixteen statistical tests in the test suite analyse sequences to identify biases, trends, or deviations from randomness. Each test operates under the null hypothesis, which assumes the input sequence is random. The significance level (α), commonly set at 0.01, establishes the threshold for randomness.

A sequence passes a test when its p-value surpasses the α value, indicating no significant deviations from randomness. This research evaluated five chaotic sequences (x, y, z, u, and v) generated by a new proposed 5D hyperchaotic system for randomness using the NIST SP 800 test suite. All sixteen tests, including the Frequency Test, Block Frequency Test, Runs Test, and others, were used to test each chaotic sequence independently. These tests evaluate several attributes, including correlation qualities, uniform distribution, and lack of patterns. The research results revealed that all five chaotic sequences passed every test in the NIST suite, with p-values consistently passing the crucial 0.01 threshold shown in Table 2. The proposed New 5D Hyper chaotic system is appropriate for cryptography and secure communication applications since it produces random and unexpected sequences.

**Table 2:** NIST test for new 5D hyperchaotic system.

S.No	Test name	P -value for new 5D hyper chaotic system				
		x	y	Z	u	v
1	Frequency test (Monobit)	**0.0201068**	**0.5314551**	**0.1463020**	**0.4607978**	**0.0189405**
2	Block frequency test	**0.5970293**	**0.1035421**	**0.6646266**	**0.6194617**	**0.5778320**
3	Run test	**0.6988461**	**0.3275552**	**0.6219753**	**0.9513798**	**0.6266024**
4	Longest run test	**0.4689894**	**0.6542549**	**0.23100262**	**0.1258558**	**0.4050830**
5	Binary matrix rank test	**0.0773196**	**0.6882664**	**0.6882664**	**0.2079514**	**0.4769277**
6	FFT	**0.6967754**	**0.2109095**	**0.9836370**	**0.4856046**	**0.6666897**
7	Non-overlapping Template	**0.4573446**	**0.0306840**	**0.6871591**	**0.8137510**	**0.6494967**
8	Overlapping template matching	**0.1165542**	**0.5476138**	**0.3275624**	**0.3246632**	**0.6453774**
9	Universal	**0.2757099**	**0.7681392**	**0.8523831**	**0.4743927**	**0.4211991**
10	Linear complexity test	**0.1341840**	**0.5165649**	**0.2206264**	**0.527770**	**0.9307552**
11	Serial test	**0.1531853**	**0.6603542**	**0.3981348**	**0.4988610**	**0.1007852**
12	Approximate entropy test	**0.0185974**	**0.3316710**	**0.0598260**	**0.0235578**	**0.1130205**
13	Cumulative sum test (Forward)	**0.0223825**	**0.7594272**	**0.1404953**	**0.7280412**	**0.0325604**
14	Cumulative sum test (Backward)	**0.0378811**	**0.665202**	**0.2803695**	**0.6037458**	**0.0190538**
15	Random Excursion	**0.9625657**	**0.0536367**	**0.4407729**	**0.1851788**	**0.1717732**
16	Random Excursion variant	**0.0133283**	**0.0386059**	**0.5186050**	**0.7394399**	**0.2852529**

### Customized U-Net architecture for segmenting images

**Fig. 5:**
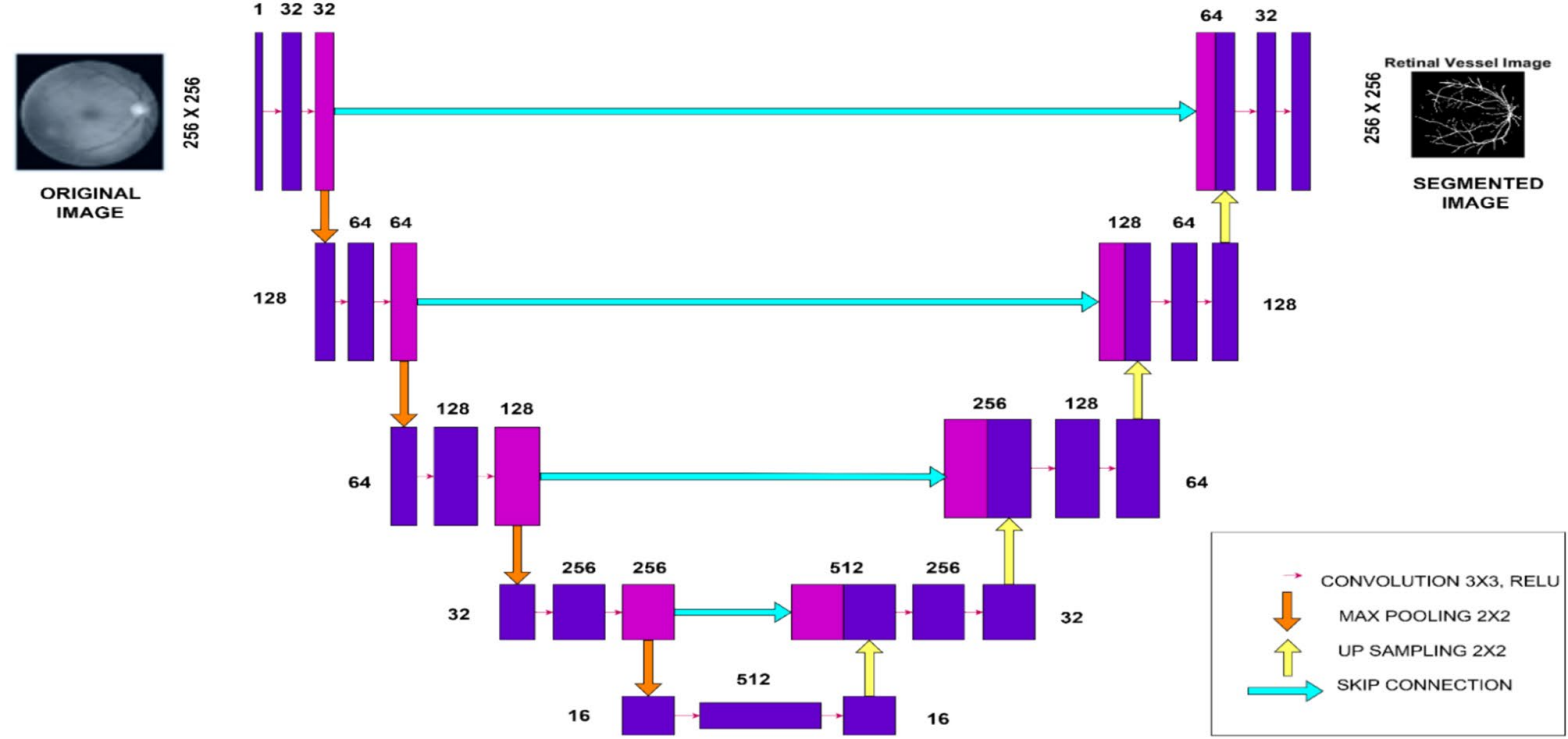
U-NET Architecture.

A convolutional neural network (CNN) architecture called U-Net was created especially for image segmentation, as shown in Fig. 5**.** It was first utilized for medical image applications^33^ but is now widely applied in many domains. An asymmetric expansive path (decoder) for reconstructing high-resolution output and a contracting path (encoder) for feature extraction make the model symmetric design. The encoder generates hierarchical features by gradually down-sampling the image using max-pooling and convolutional layers. To restore spatial resolution, the decoder, on the other hand, up-samples the feature maps and refines them using more convolutional layers. Skip connections, which connect corresponding encoder and decoder layers and assist in retaining spatial information while enhancing localisation accuracy, are a fundamental component of U-Net. The final 1 × 1 convolution maps the feature maps to the desired output classes, making it practical for binary and multi-class segmentation tasks. U-Net’s ability to perform well with limited training data and data augmentation techniques makes it a powerful tool for image segmentation in various domains^34^**,** such as medical imaging and remote sensing. In this proposed work, the U-Net architecture segments the critical regions of medical images, which are then used as initial key values to generate the new 5D hyperchaotic map. Table 3**.** presents the customised U-Net architecture, detailing the number of layers and the specific operations performed at each layer.

**Table 3:** Customized U-NET architecture description.

Operation	Specifications/Parameters	Description
Input	Gray scale medical mage −256 × 256	Medical images are spatially changed to 256 × 256 and it is normalised to [0, 1]
Encoder block 1	Double-stacked 3 × 3 convolutional layers with 64 filters, ReLU	Relu activation introduces nonlinearity in the first feature extraction from the input image
	1 × 2x2 MAX POOLING	Down samples the feature map by a factor of 2
Encoder block 2	2 × 3x3 Convolution (128 FILTERS), ReLU	Increases the depth and captures more complex features
	1 × 2x2 Max Pooling	Further reduces spatial resolution
Encoder Block 3	2 × 3x3 Convolution (256 filters), ReLU	Extracts deeper features from the image
	1 × 2x2 MAX POOLING	Further down-sampling
Bottleneck	2 × 3x3 Convolution (512 filters), ReLU	The deepest part of the network that learns the most abstract features
Decoder Block 3	1xUp-sampling (Transposed Convolution)	Begins the up-sampling process to restore spatial dimensions
	2 × 3x3 Convolution, ReLU	Refines the features after up-sampling
Decoder Block 2	1 × Up-sampling (Transposed Convolution)	Continue up-sampling the feature maps
	2 × 3x3 Convolution, ReLU	Refines and integrates features from the corresponding encoder block via skip connections
Decoder Block 1	1 × Up-sampling (Transposed Convolution)	Restores the original image resolution
	2 × 3x3 Convolution, ReLU	Combines fine details from early encoder layers with high-level features
Output	1 × 1 Convolution, Sigmoid (binary segmentation)	Maps the combined feature maps to a single-channel output, producing a segmentation mask with probabilities [0,1]

## Proposed block diagram

**Fig. 6:**
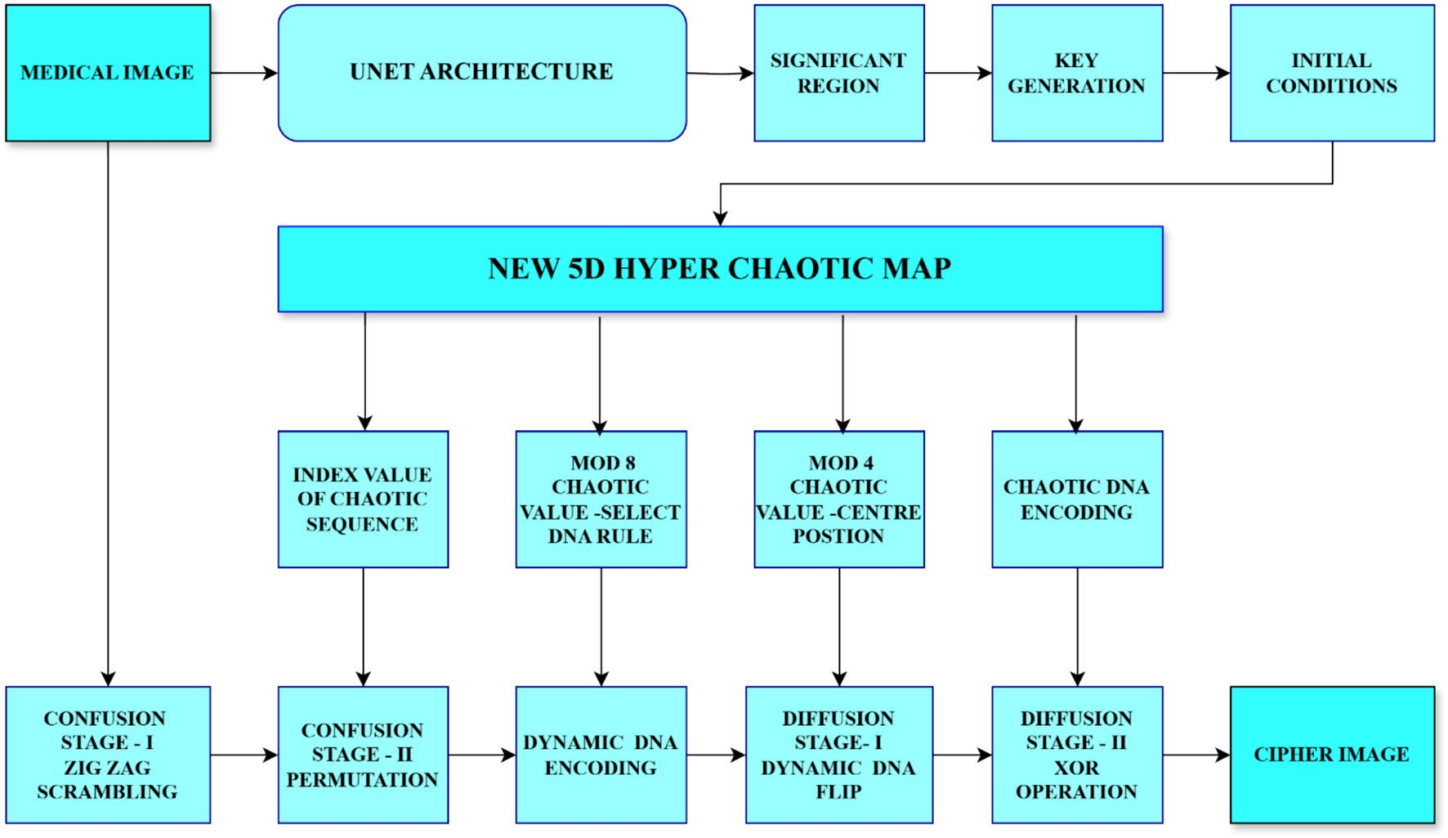
Proposed block diagram.

The block diagram in Fig. 6 illustrates an efficient medical image encryption method that combines New 5D hyperchaotic maps, U-Net architecture, and DNA-based encoding techniques to guarantee robustness and confidentiality. At the initial stage of the procedure, a U-Net architecture is used to process a medical image to extract significant regions. Critical regions set the parameters of a new five-dimensional (5D) hyperchaotic map to generate encryption keys. This chaotic map is essential to the encryption process because it generates chaotic sequences that add complexity and randomness at different points in time.

The two main stages of the encryption process are diffusion and confusion. During the confusion phase, the medical image utilizes chaotic index values, which further obfuscates the image structure with zig-zag scrambling and disturbs the spatial arrangement of pixels. The diffusion phase uses biological system-inspired dynamic DNA encoding methods. With specific mod 8 chaotic values determining the DNA rule selection and center location (mod 4) for encoding operations, chaotic sequences dynamically encode the image into DNA sequences. As a result, the image of data undergoes a more intricate and uncertain transformation. The dynamic DNA flip and an XOR operation comprise the two stages of the diffusion stage. The dynamic DNA flip uses the chaotic map’s center coordinates to scramble the DNA-encoded data further, and the XOR operation adds more diffusion by fusing the encoded image with the chaotic DNA sequence. As a result of this proposed method, the cipher image preserves highly confidential medical data and ensures the secure transfer of images. The algorithm adds the advantages of complex dynamic DNA-based encoding and the inherent randomness of chaotic systems by providing an extremely secure way to protect medical images. By highlighting significant features of the image as the key image, the U-Net architecture enhances the encryption process overall.

### Proposed algorithm

Medical images are encrypted and decrypted using an innovative 5D hyperchaotic technique. The complex key created from the crucial areas of medical images enhanced the algorithm’s performance. The new high-dimensional chaotic system resists unwanted access to the medical image and expands the chaotic range to a broader spread. The proposed algorithm protects the medical image by incorporating two shuffling and diffusion operation stages.

A medical image is resized and then converted to grayscale to simplify the data for subsequent processing. The main target of the encryption is this grayscale image. The reduced U-Net architecture’s predicted image size matches the measurements of the grayscale medical image. U-Net learns more complicated features with its deep architecture and performs segmentation tasks more accurately, mainly when dealing with more complex image structures. The algorithm generates encryption keys using statistical features derived from the pixel intensity values of the critical region of the medical image, which indicates important structural features.

In order to prove the uniqueness of our contribution, the proposed algorithm dynamically integrates the initialization of a newly derived five-dimensional hyperchaotic map that maximizes the state space complexity to per-image statistics data extracted using a simplified U-Net. These statistics of key control the zigzag scrambling, permutation and two-stage DNA diffusion (new dynamic DNA flip followed by XOR), that maximizes confusion and diffusion operations. Combining image segmentation for key generation, chaotic sequence generation, permutation, and diffusion offers improved unpredictability and resilience to attacks.

**Figure:**
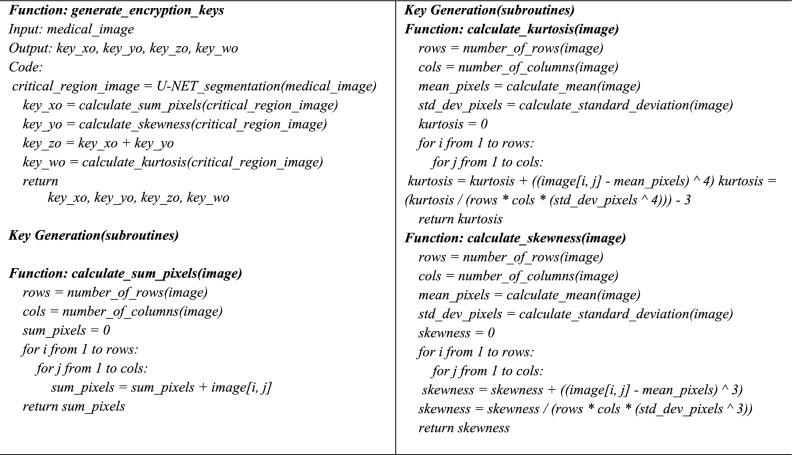
**Algorithm 1:** Key Generation

### Step 1: Key generation

The key generation process relies on a statistical analysis of the significant region in the medical image. The U-Net architecture uses different medical datasets**,** such as RITE^35^, DRIVE^36^, BRAT2020^37^, Breast Ultrasound Images Dataset^38^, GlaS (Gland segmentation dataset)^39^, and Chest x-ray dataset^40^, for extracting critical regions from the medical image. All the databases used are publicly accessible. The U-Net architecture is used to segment significant images from medical images, which are the most important part of the image. This considerable image acts as a key to the original image. Each test image has its unique key**,** which is used to encrypt the image. Four key values (key_xo, key_yo, key_zo, key_wo)) are derived from the pixel values of critical regions in the medical image and allocated as initial conditions to five chaotic sequences. These keys are crucial for initializing the new 5D Hyper chaotic system, which drives the encryption. The statistics of the critical regions are derived from the Eqs. (4–6)

Key ${\mathbf{K}}_{\mathbf{x}}$: Sum of all pixel values, captures the overall brightness or intensity of the extracted (significant) image.

4 $${k}_{x }= {\sum }_{I=1}^{R}{\sum }_{J=1}^{C}{I}_{1}\left(i,j\right)$$

where:

- ${\text{I}}_{1}\left(\text{i},\text{j}\right)$ is the pixel intensity at position (i,j),
- R and C are the dimensions of the image.
- R—Refers to the number of rows in the image.
- C—Refers to the number of columns in the image.
- Key ${\text{K}}_{\text{x}}$ : Sum of all Pixel Values

Measures the pixel intensity distribution asymmetry, indicating variations in contrast and structure.

5 $${K}_{y}= \frac{1}{M\times N} {\sum }_{I=1 }^{R}{\sum }_{J=1 }^{C} {\left(\frac{I\left(i,j\right)-\text{E}}{{\sigma }_{a}}\right)}^{3}$$

where:

- E is the mean pixel value
- ${\upsigma }_{\text{a}}$ is the standard deviation of pixel values.
  - Key ${\text{K}}_{\text{y}}$: Skewness of Pixel Values

Key ${\mathbf{K}}_{\mathbf{z}}$: Sum of ${{\varvec{K}}}_{{\varvec{x}}}$ and ${{\varvec{K}}}_{{\varvec{y}}}$

Combines overall intensity and skewness, providing a value reflecting brightness and contrast.

$${K}_{Z}= {K}_{x }+ {K}_{y}$$

Key ${\mathbf{k}}_{\mathbf{w}}$: Kurtosis of pixel values

Indicates the concentration of pixel values around the mean, capturing the sharpness or flatness of the intensity distribution.

6 $${K}_{w}= \frac{1}{M\times N} {\sum }_{I=1 }^{R}{\sum }_{J=1 }^{C} {\left(\frac{I\left(i,j\right)-\text{E}}{{\sigma }_{a}}\right)}^{4}-3$$

The subtraction of 3 adjusts for a normal distribution baseline, making it easier to interpret kurtosis relative to standard intensity distributions. These key values are normalised to fall within a suitable range for chaotic sequence generation. The initial condition for the chaotic sequence is allocated as xo = key_xo, yo = key_yo, zo = key_zo, uo = key_wo and vo = key_zo. After normalization, the initial conditions for one test image, such as the Drive test image-1, obtained from the segmented image of U-Net are xo = −0.5, and yo = −1.5, zo =—0.5 and uo = −1.5, vo = −0.5 Similarly, we extracted the initial conditions of the chaotic map for each test image from the critical region of corresponding medical images segmented by U-Net architecture.

### Motivation for employing U-net-based segmentation:

U-Net is a comparatively reduced-overhead model with fast execution and provides a balanced architecture that minimizes the trade-off between data security and resource consumption. U-Net is a lightweight model that still achieves segmentation accuracy similar to more complex neural networks. U-Net is well suited technique for real-time encryption, ensuring that system resilience and encryption key computation accuracy are sustained without demanding much computing power.

For comparison of U-Net with different models, we use the Adam optimizer with the binary cross-entropy loss (fit for binary masks) for image segmentation. To prevent overfitting, the training is performed up to 200 epochs with early stopping based on validation loss with patience of ~ 10 epochs). Eight batches are used, and 20% of the training data is divided for validation The parameters are used to validate the performance of the U-Net network, and the performance comparison of the networks is given in Table 4.

**Table 4:** Performance comparison of neural networks.

Model	Accuracy	Precision	Recall	FI-score	IOU	Dice coefficient
U-NET	**0.9606**	**0.8046**	**0.7547**	**0.7789**	**0.6821**	**0.8110**
ResU-Net	**0.9594**	**0.8232**	**0.6770**	**0.7430**	**0.5910**	**0.7430**
Attention U-Net	**0.9835**	**0.8634**	**0.8012**	**0.8314**	**0.8384**	**0.9014**

Accuracy:

Accuracy is the ratio of actual positive pixels from the background and foreground pixels to the total number of pixels

7 $$\text{Accuracy}= \frac{TP+TN}{TP+TN+FP+FN}$$

Precision (Positive Predictive Value):

The proportion of detected positive foreground pixels (predicted positive pixels).

8 $$\text{Precision }= \frac{TP}{(TP+FP)}$$

Recall (Sensitivity or True Positive Rate):

The metric measures the proportion of ground truth positive pixels correctly predicted out of all positive classes.

9 $$\text{Sensitivity }(\text{Recall}) = \frac{TP}{(TP+FN)}$$

F1-Score:

The harmonic mean of Precision and Recall. It balances the trade-off between the precision and recall parameters.

10 $$\text{F}1\_\text{Score}=2\times \frac{Precision \times Recall}{precision+Recall}$$

IoU (Intersection over Union):

Overlap between predicted and ground truth segments divided by their union.

11 $$\text{Intersection over Union }(\text{IoU}) = \frac{TP}{TP+FP+FN}$$

Dice Coefficient:

Measures similarity between predicted and ground truth segments.

12 $$\text{Dice coefficient }= \frac{2TP}{2TP+FP+FN}$$

Here, TP, TN, FP, and FN refer to the counts of true-positive, true-negative, false-positive, and false-negative pixels on the test data set. Dice and IoU capture region overlap, while Precision/Sensitivity captures pixel-wise accuracy. All metrics are computed on the final predicted binary versus ground truth masks.

As Table 4 shows that U-Net achieves 0.9606 accuracy and a 0.8110 Dice score. It is marginally below Attention U-Net (0.9835/0.9014) and significantly ahead of ResU-Net (0.9594/0.7430). Still, U-Net achieves the optimum balance between the quality of segmented data and computing efficiency for real-time encryption by using fewer parameters and inferring data 25–35% faster.

### segmentation‐key robustness:

Segmentation accuracy directly impacts the chaotic sequence of encryption since the encryption key is generated from the segmented region. We use the following measures to reduce the likelihood of segmentation errors:

- The segmentation output is consistent across samples because of early Stopping, which is based on validation loss and chooses the most generalizable U-Net weights.
- Morphological Processing is applied to the predicted segmentation mask to suppress distortion and artifacts and ensure a better match with the ground truth region. The lightweight operation enhances privacy and robustness against attacks.
- Validation metrics, such as IoU or Dice coefficient, are used to determine the quality of segmented regions and detect the degradation of segmented data on a validation dataset. If the segmentation probability falls below a threshold, the encryption process is retrained with a updated weights and corrected mask.

These security measures maintain reliability and safety by ensuring the encryption procedure is closely linked to precise and verifiable segmentation.

**Figure:**
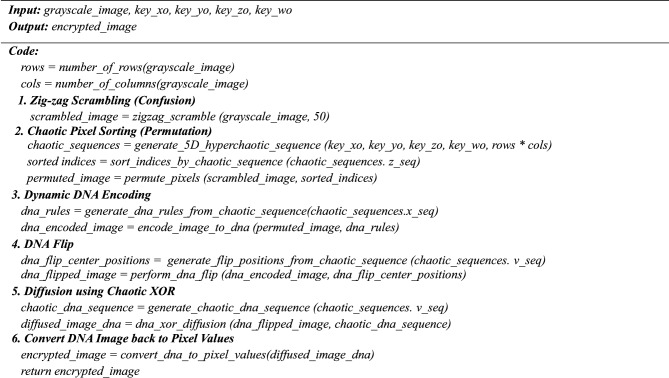
**Algorithm 2:** Function: encrypt_medical_image

### Step 2: Zig-zag Scrambling (Confusion Phase)

**Fig. 7:**
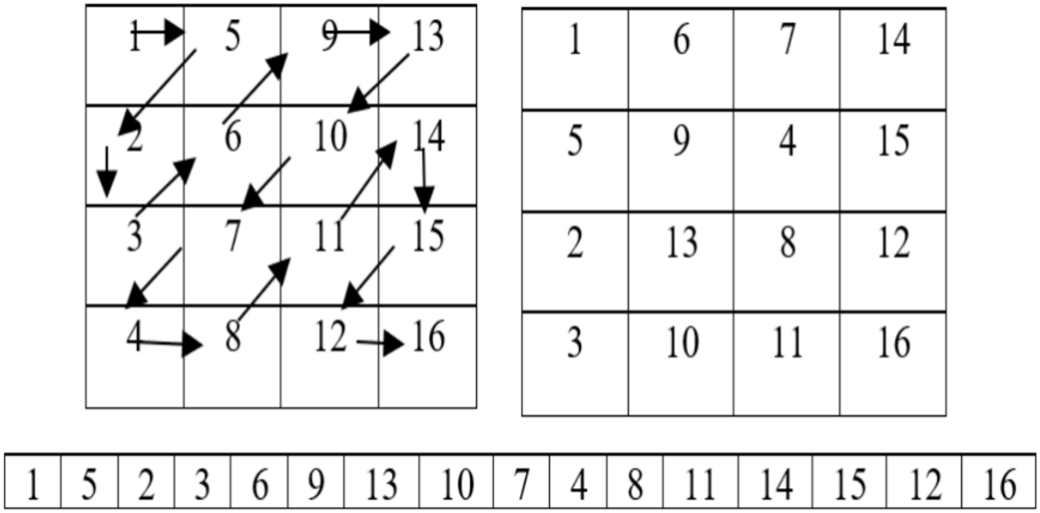
Zig zag scrambling.

The first stage of encryption, known as the confusion phase, begins with conventional zig-zag scrambling^41^**.** The grayscale image is rearranged in this process according to a zig-zag pattern, reorganising the pixels non-linearly. Zig-zag scrambling is applied iteratively (in this case, 50 iterations) to increase the randomness in the pixel arrangement further. The zig-zag pattern is generated by traversing the image in a diagonal zig-zag order shown in Fig. 7, beginning at the top-left corner and moving alternately up and down. This transforms the image’s pixel arrangement into a new order, creating structural confusion. After several iterations, the scrambled image no longer resembles the source image, making it difficult for an unauthorized person to discern the original data.

**Figure:**
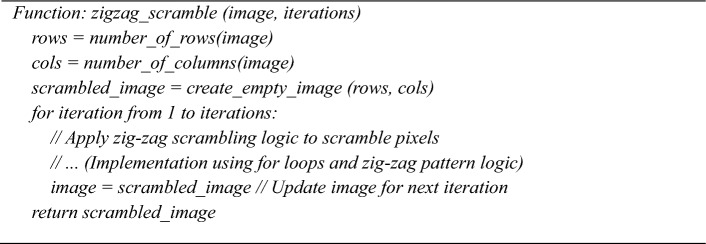
**Algorithm 3:** Zigzag_scramble (image, iterations)

**Figure:**
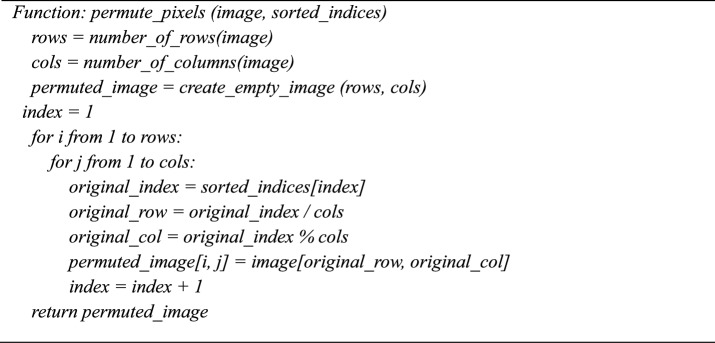
**Algorithm 4:** Permute_pixels (image, sorted_indices)

### Step 3: Chaotic pixel sorting (Permutation)

Next, zig-zag scrambled image pixels are further scrambled using chaotic values from the Z variable of the New 5D hyperchaotic system. The pixel values are arranged based on the chaotic sequence generated by Z. This process, known as chaotic pixel sorting, enhances the confusion by introducing an additional layer of randomness. Pixels are reorganized according to their association with the chaotic sequence, concealing any remaining structural patterns in Eq. (13).

13 $${C}_{1}=I \left(Sort \left(Z\right)\right)$$

where

- ${\text{C}}_{1}$ is the confused image.
- I is the zig-zag image
- $\text{Sort }\left(\text{Z}\right)$ provides the new arrangement based on the sorted indices.

### Step 4: Dynamic DNA encoding

Four nucleic acid bases make up a DNA sequence: A (adenine), G (guanine), C (cytosine), and T (thymine). C and G are complementary, and A and T are complementary. Two bits, 01, 10, 00, and 11, are typically used to encode the four DNA bases A, C, G, and T. Since 0 and 1 are complementary in binary encoding, so are 00 and 11 and 01 and 10. There are 24 encoding rules when 01, 10, 00, and 11 are encoded using the four bases C, G, A, and T. Of these, only eight DNA rules shown in Table 3**.** meet the complete relations among the complementary bases.

In the next phase, diffusion is performed by converting the encrypted image from the first phase into a DNA image^42^**,** using one of the chaotic sequences as the DNA rule. The hyperchaotic sequence is generated and converted into the DNA rules from 1 to 8 for each pixel value in the encrypted image. Based on the DNA rule number^43^**,** each pixel in the confused image is converted into a four-base DNA variable.

14 $${E}_{DNA}\left(i,j\right)=DN{A}_{Encode}\left( {C}_{1}\left(i,j\right),{R}_{1}\left(i,j\right)\right)$$

where

- ${C}_{1}\left(i,j\right)$ is pixel value at (i, j) in the Confused image,
- ${\text{R}}_{1}\left(\text{i},\text{j}\right)$ is DNA rule derived from the 5D chaotic sequence.
- ${\text{E}}_{\text{DNA}}\left(\text{i},\text{j}\right)$ is the resulting DNA sequence for the confused image at (i,j)
- ${\text{DNA}}_{\text{Encode}}$ is function mapping pixel values to DNA sequences using ${\text{R}}_{1}$ (i,j).

**Figure:**
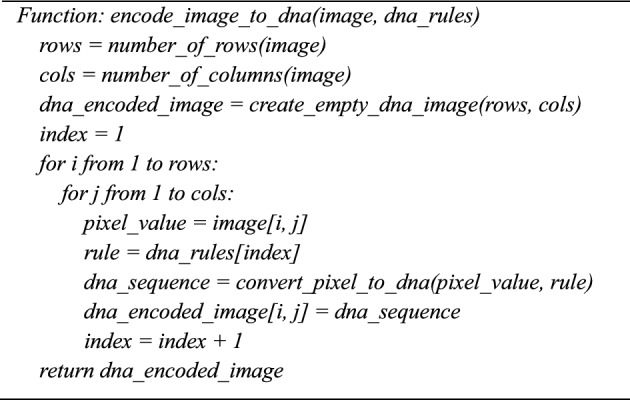
**Algorithm 5:** Encode_image_to_dna (image, dna_rules).

**Figure:**
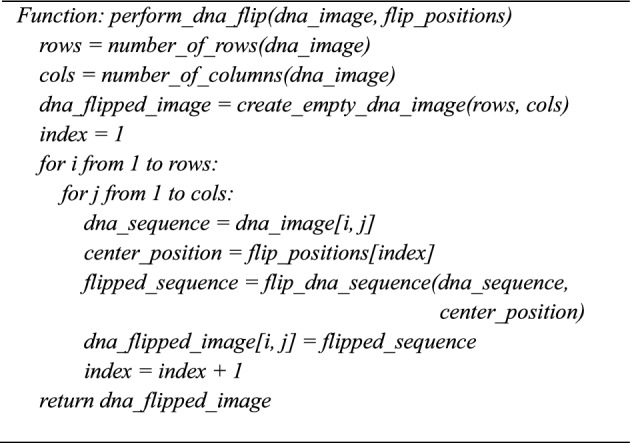
**Algorithm 6:** Perform_dna_flip(dna_image, flip_positions)

### Step 5: DNA Flip

Based on the chaotic value, the dynamic DNA flip is performed from the previous stage’s output, which is given in Eqs. (15-17). The hyperchaotic sequence V is converted into a mod 4 value, which gives the center position to the DNA image obtained from the previous step to swap each position of DNA value from left to right of the sequence and right to left of the sequence.

15 $${P}_{1}\left(i,j\right)=\left(\left[{V}_{1}\left(i,j\right) X {10}^{\begin{array}{c}26 \\ \end{array}}\right]mod 4\right)+1$$

where is ${\text{P}}_{1}\left(\text{i},\text{j}\right)$ center position for the DNA swap (values 1 to 4,${\text{V}}_{1}\left(\text{i},\text{j}\right) is$ hyperchaotic value at pixel (i, j)

16 $${E}_{DNA}\left(i,j\right)=\left[{B}_{1},{B}_{2},{B}_{3},{B}_{4}\right]$$

${\text{E}}_{\text{DNA}}\left(\text{i},\text{j}\right) :$ DNA value for pixel (i, j) with four DNA bases $\left[{\text{B}}_{1},{\text{B}}_{2},{\text{B}}_{3},{\text{B}}_{4}\right]$

17 $${D}_{DNA}{\prime}= \left\{\begin{array}{c}\left[{B}_{2},{B}_{3},{B}_{4},{B}_{1}\right] ,P\left(i,j\right)=1 \\ \left[{B}_{3},{B}_{4},{B}_{2},{B}_{1}\right] ,P\left(i,j\right)=2\\ \left[{B}_{4},{B}_{3},{B}_{1},{B}_{2}\right],P\left(i,j\right)=3\\ \left[{B}_{4},{B}_{1},{B}_{2},{B}_{3}\right],P\left(i,j\right)=4\end{array}\right.$$

where $D^{\prime}_{DNA}$ is swapped DNA sequence for pixel (i, j).

### Step 6: Diffusion using hyper chaotic XOR (Diffusion Phase)

In the second stage of the diffusion operation, the pixel values are altered using bitwise XOR operations in conjunction with.

chaotic values from the novel 5D Hyper chaotic system. In this process, the chaotic sequence ‘V’ from the hyperchaotic system is converted into 8-bit binary values. The DNA of the V-binary values is then XORed with the pixel values of the DNA Flip image. This XOR diffusion technique effectively spreads the impact of any single change throughout the entire image, ensuring that even minor changes in the input result in significant variations in the output, thereby enhancing security. The diffusion image is generated by applying the DNA XOR^44^ operation to the DNA flip image and chaotic sequence- ‘V’ DNA image. Table 5**.** and Table 6. show the DNA rule and DNA XOR.

18 $${V}_{DNA }\left(i,j\right)=DN{A}_{Transform} \left(Bin\left(V\left(i,j\right)\right)\right)$$

where $\text{V}\left(\text{i},\text{j}\right)$ is the chaotic value at (i,j),$\text{Bin}$ () is conversion of V(i,j)) to an 8-bit binary value. DNA_TRANSFORM_() is the conversion of binary to a DNA sequence.${\text{V}}_{\text{DNA }}\left(\text{i},\text{j}\right)$ is the resulting DNA sequence for V image.

19 $$Cipher Image \left(i,j\right)= {D}_{DNA}{\prime} \left(i,j\right)\oplus {V}_{DNA}\left(i,j\right)$$

where Cipher Image(i,j)$\text{is}$ pixel value at (i, j) in the diffused image in DNA format.

**Table 5:** DNA rule.

Dna rule no	00	11	10	01
1	**A**	**T**	**G**	**C**
2	**A**	**T**	**C**	**G**
3	**T**	**A**	**G**	**C**
4	**T**	**A**	**C**	**G**
5	**G**	**T**	**A**	**C**
6	**G**	**T**	**C**	**A**
7	**C**	**T**	**A**	**G**
8	**C**	**T**	**G**	**A**

**Table 6:** DNA XOR table for the proposed algorithm.

DNA base1	DNA base 2	XOR Result
A	**A**	**A**
A	**T**	**T**
A	**G**	**G**
A	**C**	**C**
T	**A**	**T**
T	**T**	**A**
T	**G**	**C**
T	**C**	**G**
G	**A**	**G**
G	**T**	**C**
G	**G**	**A**
G	**C**	**T**
C	**A**	**C**
C	**T**	**G**

**Fig. 8:**
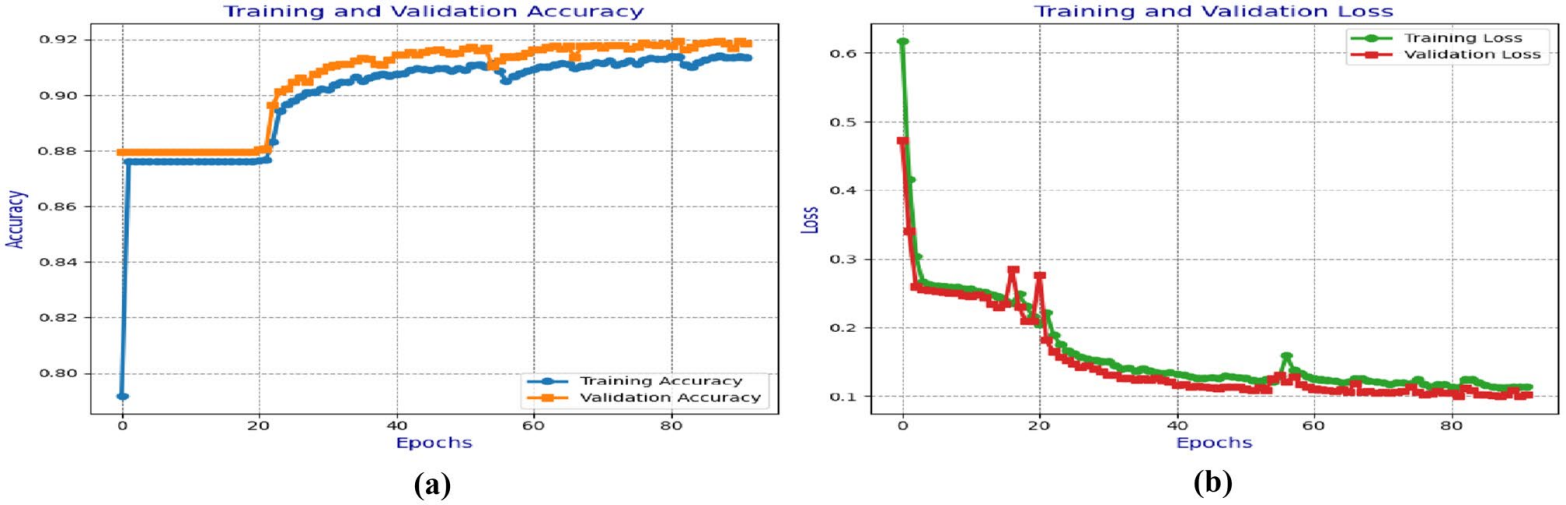
(**a**) Training and validation accuracy of the U-NET network for the RITE dataset (**b**) Training and validation loss for the RITE dataset.

As steps 5 and 6 explain, the proposed 5D hyperchaotic sequence controls the two diffusion layers. Each pixel value of permuted image is encoded into DNA bases according to the chaotic sequence. Then, it is rotated by a per-pixel index based on the chaos mod four value, guaranteeing that every neighboring pixel exhibits different flip patterns. In stage II (step 6), another chaotic sequence is converted into DNA and XOR-ed with the flipped image individually on each DNA base. Thus, cascading stages of DNA flip diffusion with DNA XOR-ed ensure that even a one-bit change in the source image or the secret key disperses nonlinearly and chaotically across the entire encrypted image, achieving strong pixel dependency and a robust avalanche effect across the entire encrypted image. The simulation results show that NPCR > 99.6% and UACI > 33%, ensuring an intense avalanche effect and demonstrating the security strength of our diffusion process against differential attacks.(Fig. 8).

**Figure:**
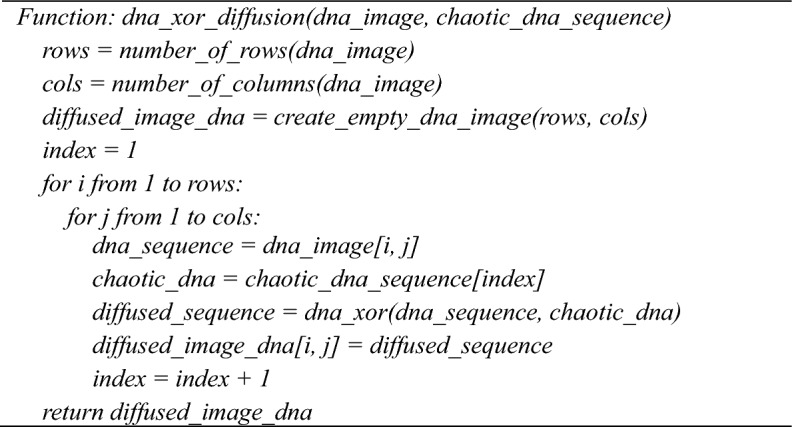
**Algorithm7:** dna_xor_diffusion(dna_image, chaotic_dna_sequence)

**Figure:**
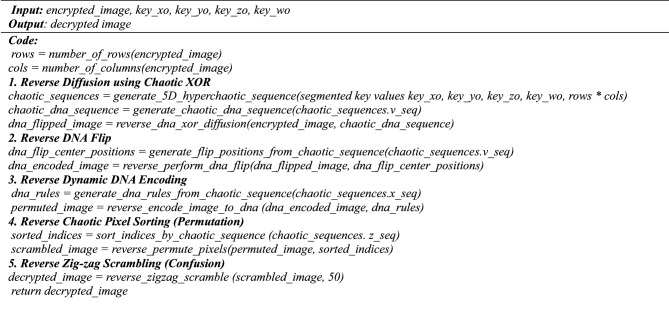
**Algorithm 8:** Function: decrypt_medical_image

## Experimental results

**Fig. 9:**
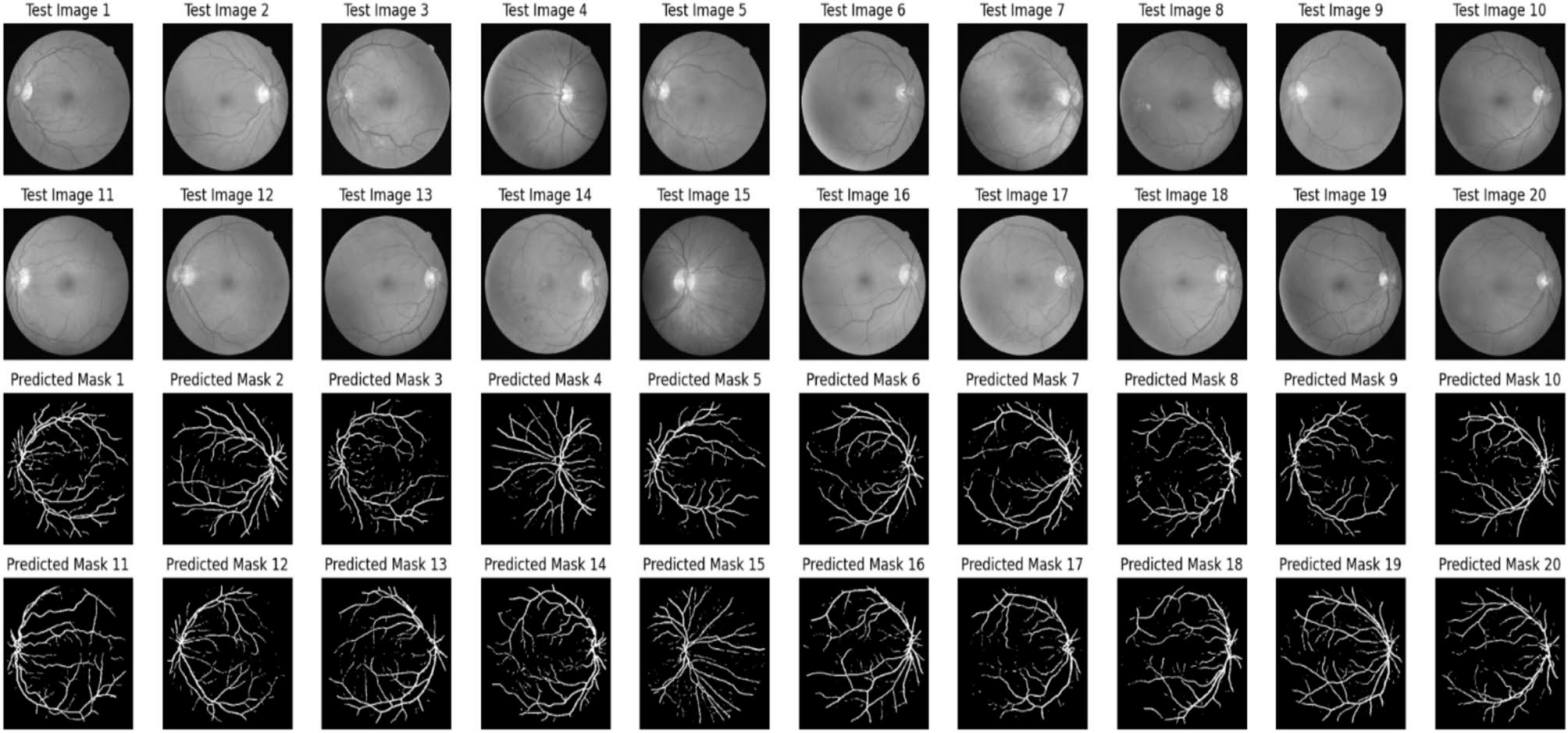
Drive data set -Test image with predicted retinal Image from U-NET.

**Fig. 10:**
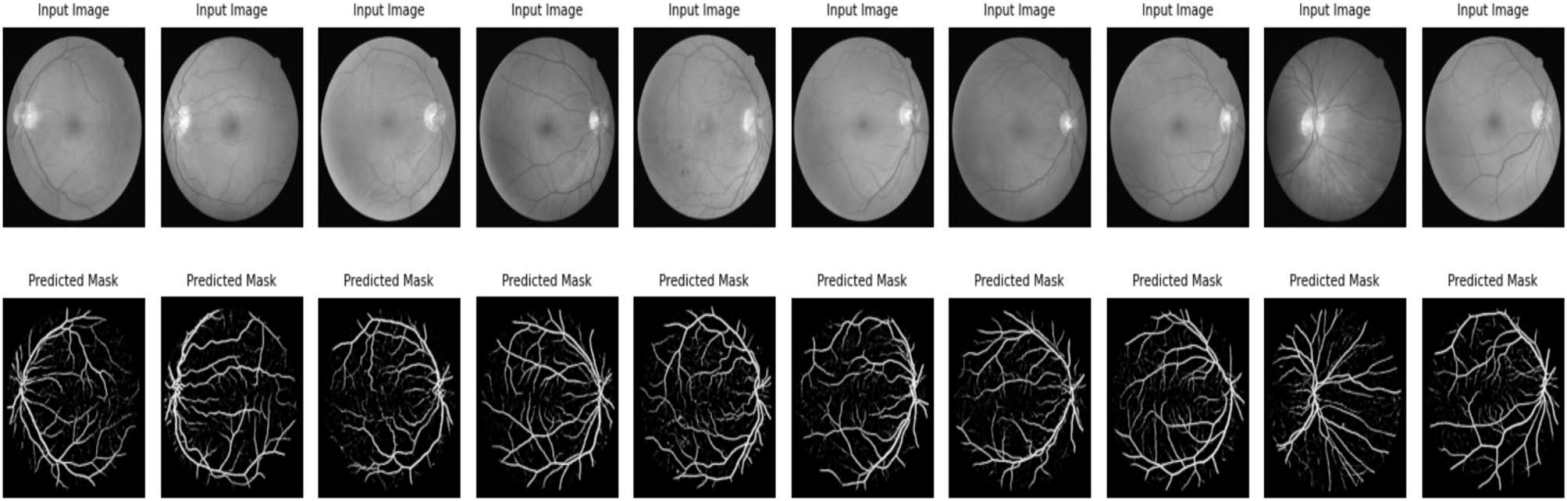
Rite data set -Test image with extracted retinal Image from U-NET.

**Fig. 11:**
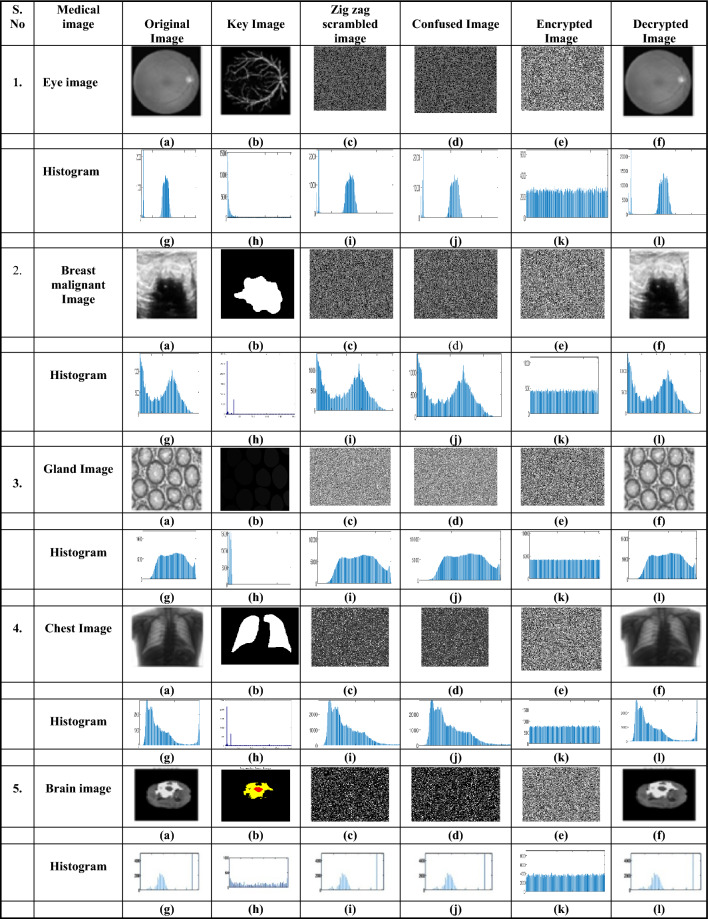
(**a**)original image, (**b**) key image, (**c**) zig-zag scrambled image, (**d**)confused image, (**e**) encrypted image (**f**) decrypted image (**g**) original image histogram, (**h**) key image histogram, (**i**) zig-zag scrambled image histogram, (**j**) confused image histogram, (**k**) encrypted image histogram, and (**l**) decrypted image histogram.

**Fig. 12:**
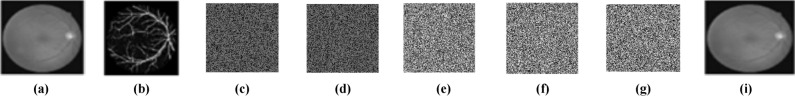
(**a**) original eye image, (**b**) predicted retinal image, (**c**) zig-zag scrambled image, (**d**) confused image, (**e**) DNA swapped image (**f**) chaotic sequence ‘V’-DNA image, (**g**), DNA XOR image (Final cipher image) and (**i**) decrypted image.

The U-Net model trains on different medical images to extract critical regions, focusing on areas of interest. Figure 9 and Fig. 10 illustrate the U-Net architecture in action, showcasing the extraction of retinal vessels from original eye images. The U-Net architecture utilizes the RITE dataset from the various source images to extract the retinal vessels from the images. The model uses the RITE dataset, containing 80 training images, to predict retinal features accurately. The U-Net processes 10 test images from the RITE dataset and 20 from the DRIVE dataset during testing. By extracting retinal vessels, the U-Net model highlights significant details in each eye image, which play a crucial role in subsequent analyses. Figure 8 displays the accuracy and loss of the segmenting operations applied to a RITE test image. Figure 11 displays the step-by-step approach of the encryption technique with its corresponding histogram.Fig. 12 shows the two confused stage and two diffused stage outputs with original and decrypted images.

## Performance analysis

### Statistical analysis

#### Correlation analysis

**Fig. 13:**
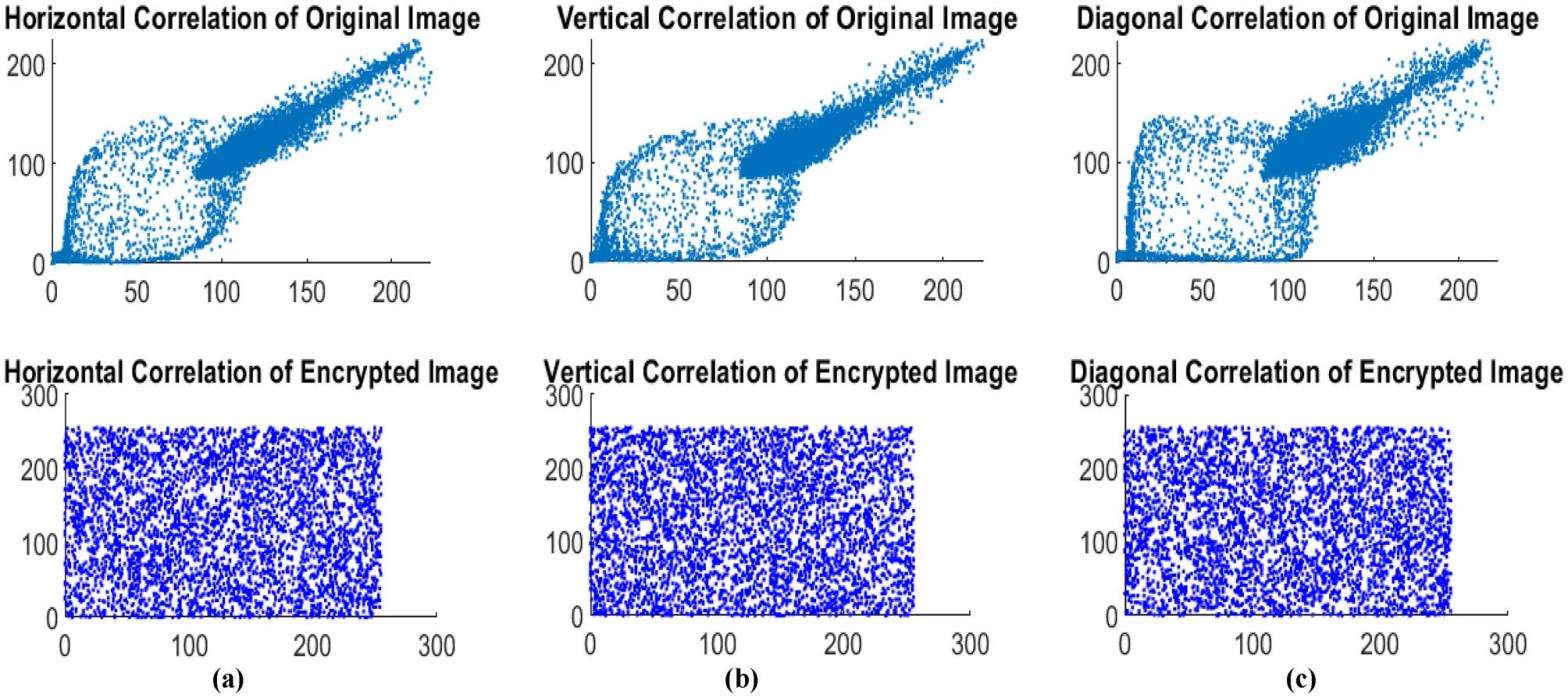
(**a**) Horizontal correlation of original image and encrypted image, (**b**) vertical correlation of original image and encrypted image, and (**c**) Diagonal correlation of original image and encrypted image for Medical Drive test image_1.

Correlation analysis evaluates how effectively encryption disrupts patterns between neighboring pixels. Natural images, like face or retinal scans, often show high correlations due to similarities in structure and brightness. Effective encryption reduces these correlations to near zero, randomizing pixel values and breaking structural patterns. The proposed algorithm prevents attackers from extracting meaningful information from the cipher image. Figure 13 shows the correlation analysis for the original test eye image 1 and its encrypted version.

The correlation coefficient is essential for assessing an encryption algorithm’s security. An encrypted image with a low correlation indicates that the algorithm has successfully eliminated the relationships between nearby pixels, enhancing encryption security. The correlation coefficient r_xy_^45^ between two adjacent pixels, X_k_ and Y_k_ is calculated as

20 $${r}_{xy}= \frac{\sum_{K=1}^{N}\left({X}_{k}-{E}_{X}\right)\left({Y}_{k}-{E}_{Y}\right)}{\sqrt{{\sum_{K=1}^{N}\left({X}_{k}-{E}_{X}\right)}^{2}\sum_{K=1}^{N}{\left({Y}_{k}-{E}_{Y}\right)}^{2}}}$$

where X_k_ and Y_k_ are intensity values of adjacent pixels (for example, horizontally, vertically, or diagonally neighboring pixels), E_X_ and E_Y_ are the mean intensities of the sets of pixels X_k_ and Y_k,_ respectively and k refers to the total count of pairs in the image.

Table 7. shows the correlation coefficient value for the medical test image and the encrypted image in the horizontal, vertical, and diagonal directions. The efficacy of the encryption technique is indicated by the reduced correlation coefficient value, which is close to zero when comparing the values of the original test image.

**Table 7:** Correlation coefficient value for RITE, DRIVE test images and other medical images.

Image	Horizontal correlation coefficient		Vertical correlation coefficient		Diagonal correlation coefficient	
Drive test image	Test image	Encrypted image	Test image	Encrypted image	Test image	Encrypted image
Rite_test_01	**0.9952**	**−0.007082**	**0.9931**	**−0.002229**	**0.98627**	**0.002396**
Rite_test_02	**0.9946**	**−0.003340**	**0.9904**	**0.011874**	**0.98275**	**−0.006118**
Rite_test_03	**0.9942**	**0.004887**	**0.9909**	**−0.002266**	**0.98386**	**0.002593**
Rite_test_04	**0.9948**	**−0.007218**	**0.9901**	**−0.003637**	**0.98099**	**0.001716**
Rite_test_05	**0.9971**	**−0.000391**	**0.9936**	**−0.002719**	**0.98889**	**0.007835**
Rite_test_06	**0.9943**	**0.000616**	**0.9899**	**−0.004342**	**0.98195**	**−0.002745**
Rite_test_07	**0.9925**	**−0.003821**	**0.9878**	**0.006066**	**0.9786**	**−0.001618**
Rite_test_08	**0.9947**	**−0.002498**	**0.9908**	**−0.003725**	**0.98349**	**−0.001367**
Rite_test_09	**0.9922**	**−0.003439**	**0.9894**	**−0.002049**	**0.98049**	**0.002163**
Rite_test_10	**0.9946**	**0.003848**	**0.9912**	**0.001317**	**0.98348**	**0.002630**
Drive_test_01	**0.9944**	**−0.004510**	**0.9910**	**0.004100**	**0.98399**	**0.000327**
Drive_test_02	**0.9961**	**−0.001893**	**0.9929**	**0.000272**	**0.98736**	**0.000365**
Drive_test_03	**0.9954**	**−0.006763**	**0.9898**	**0.002406**	**0.98245**	**−0.004235**
Drive_test_04	**0.9945**	**−0.000945**	**0.9918**	**−0.004112**	**0.98237**	**−0.000316**
Drive_test_05	**0.9963**	**−0.005323**	**0.9926**	**0.001831**	**0.98629**	**−0.001713**
Drive_test_06	**0.9922**	**0.003019**	**0.9887**	**0.001550**	**0.98018**	**−0.002259**
Drive_test_07	**0.9958**	**−0.004700**	**0.9931**	**0.003148**	**0.98657**	**0.003177**
Drive_test_08	**0.9954**	**−0.005883**	**0.9930**	**−0.003347**	**0.98581**	**−0.003502**
Drive_test_09	**0.9947**	**0.001353**	**0.9914**	**0.001287**	**0.98476**	**0.001007**
Drive_test_10	**0.9959**	**0.002913**	**0.9926**	**0.002279**	**0.98707**	**−0.003756**
Drive_test_11	**0.9957**	**0.000660**	**0.9940**	**0.010806**	**0.9878**	**−0.005441**
Drive_test_12	**0.9951**	**0.003489**	**0.9916**	**0.007634**	**0.98458**	**−0.000378**
Drive_test_13	**0.9947**	**0.000741**	**0.9922**	**−0.005448**	**0.98572**	**0.001748**
Drive_test_14	**0.9952**	**−0.001414**	**0.9914**	**−0.003367**	**0.98311**	**0.006277**
Drive_test_15	**0.9974**	**0.001109**	**0.9943**	**−0.008311**	**0.99001**	**−0.003844**
Drive_test_16	**0.9948**	**−0.002163**	**0.9913**	**0.000310**	**0.98397**	**0.004270**
Drive_test_17	**0.9931**	**0.002348**	**0.9894**	**−0.000803**	**0.9809**	**0.009133**
Drive_test_18	**0.9952**	**0.004147**	**0.9920**	**−0.000568**	**0.98536**	**0.000645**
Drive_test_19	**0.9928**	**0.002006**	**0.9906**	**0.001677**	**0.98232**	**−0.000057**
Drive_test_20	**0.9951**	**−0.003326**	**0.9924**	**0.001637**	**0.98538**	**0.005711**
Breast Image	**0.9955**	**−0.002098**	**0.9841**	**−0.000279**	**0.98267**	**−0.001752**
Gland Image	**0.9911**	**−0.000809**	**0.9952**	**0.000887**	**0.98819**	**−0.002699**
Chest Image	**0.9984**	**−0.000131**	**0.9962**	**0.000573**	**0.99545**	**−0.000186**
Brain Image	**0.9471**	**−0.004920**	**0.9525**	**−0.004379**	**0.89869**	**−0.011123**

#### Histogram analysis

**Fig. 14:**
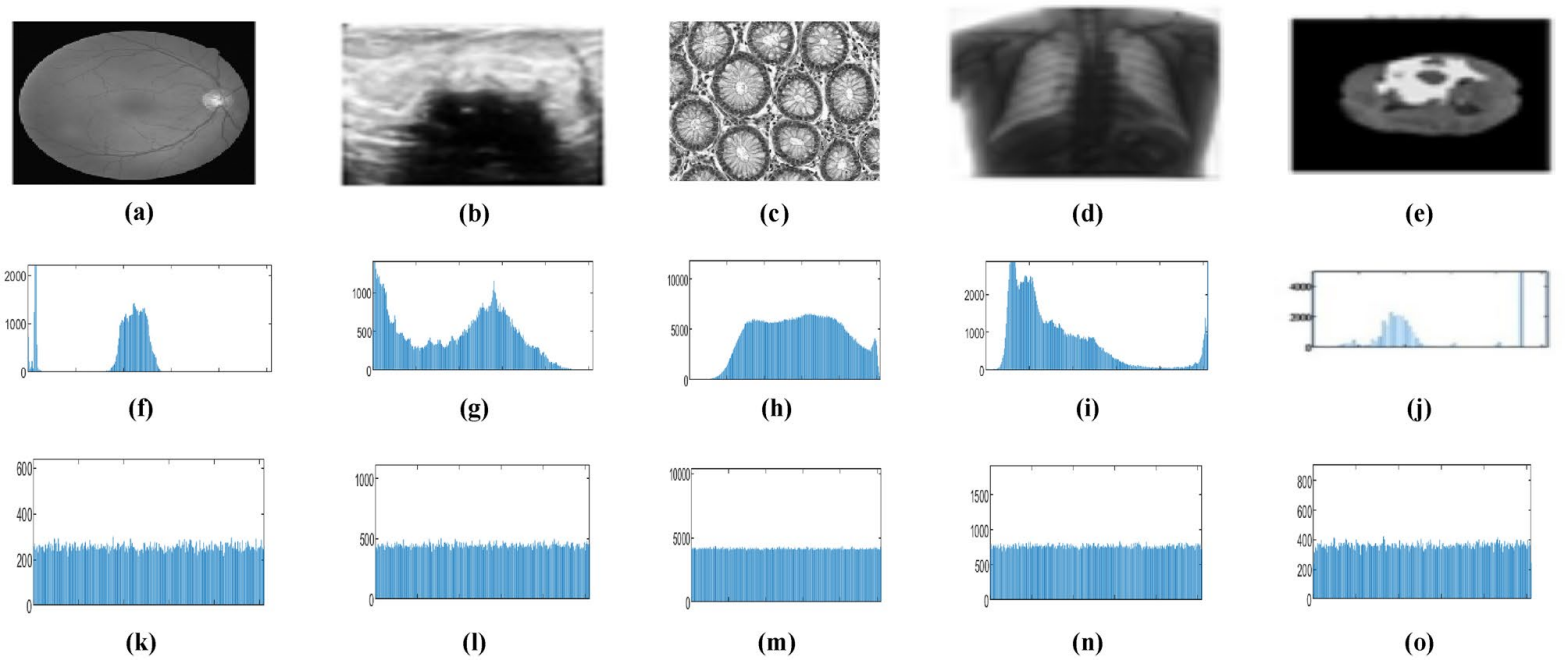
Histogram analysis of medical images. (**a**), (**b**), (**c**), (**d**), (**e**) are the 5 test medical images and (**f**), (**g**), (**h**),(**i**),(**j**) the histogram of the input medical images, (**k**), (**l**), (**m**), (**n**), (**o**) are the histogram of encrypted medical images.

By achieving an even distribution of pixel intensities, the algorithm guarantees a uniform histogram^46^in the encrypted image, effectively hiding patterns and defenses against statistical attacks. Because of this uniformity, which removes obvious connections in pixel values, attackers cannot deduce any information about the original image. Figure 14 shows the histogram of medical images and the encrypted images.

### Random test analysis

#### Chi-square test

The Chi-Square test^47^ is a statistical technique to assess how well observed frequency distributions match expected ones. It is commonly applied to determine whether a dataset is random. In the context of image encryption, the Chi-Square test examines the arrangement of element values in protected images to ensure they appear random and lack discernible patterns. The chi-square test formula given in the equation

21 $${\chi }^{2}= \sum_{i=1}^{255}\frac{ {\left({O}_{i}- {E}_{i }\right)}^{2}}{{E}_{i}}$$

where ${\text{O}}_{\text{i}}$ = Observed frequency of pixel intensities and ${\text{E}}_{\text{i}}$= Expected frequency (assuming a uniform distribution).

##### Hypothesis:

Null Hypothesis (H_0_): The observed pixel intensities follow a uniform distribution.

Alternative Hypothesis (H_1_): The observed pixel intensities deviate significantly from the uniform distribution.

Degrees of Freedom:

22 $$df=K-1$$

where k is the number of intensity scales (e.g., 256 for 8-bit grayscale images).

### Decision rule:

The calculated χ2 value is compared with the critical value from the Chi-Square dispersion table at a given P- value, the significance level (α = 0.05 or α = 0.01) :

χ2 > critical value: Reject H_0_, indicating a non-uniform distribution.

χ2 ≤ critical value: Fail to reject H_0_, indicating a uniform distribution.

A low Chi-Square value suggests a uniform distribution of pixel intensities compared to critical values at conventional significance levels (5% or 1%). The chi-square value is given in Table 8. This test helps to validate the security of encryption techniques by making encrypted images resistant to statistical attacks. The algorithm consistently produces Chi-Square values below the critical threshold for all examined medical images.

**Table 8:** Chi-square test analysis.

Medical images	Chi-square value	Critical value	
		5% = 293.2478	1% = 310.4574
Eye image	266.6172	PASS	PASS
Breast Image	238.7999	PASS	PASS
Gland Image	304.1504	PASS	PASS
Chest Image	292.4817	PASS	PASS
Brain Image	277.9814	PASS	PASS

### Potential attack analysis

#### Cropping attack analysis

**Fig. 15:**
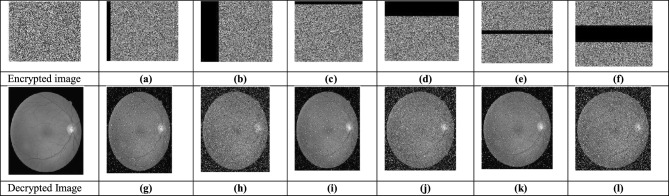
Cropping Images (**a**) and (**b**) 5% and 25% vertical cropping image, (**c**) and (**d**) 5% and 25% Horizontal cropping image, (**e**) and (**f**) 5% and 25% center cropping. (**g**) and (**h**) decrypted image of (**a**) and (**b**), (**i**) and (**j**) decrypted image of (**c**) and (**d**), and (**k**) and (**l**) decrypted image of (**e**) and (**f**).

Cropping analysis evaluates encryption^48^ resilience by testing how well the original image recovers after removing portions of the encrypted images. In this study, researchers cropped the encrypted image by 5% and 25% in lateral, perpendicular, and middle directions. The results demonstrated that the encryption method maintains robustness, enabling visible recovery even after significant cropping. Central cropping assessed data protection in the image’s middle, while horizontal and vertical cropping impacted edge content. Figure 15 shows the cropping analysis and corresponding decrypted images. Increasing the cropping percentages introduced more distortion, but the encryption method successfully preserved essential image content despite partial data loss.

##### Noise attack analysis

**Fig. 16:**
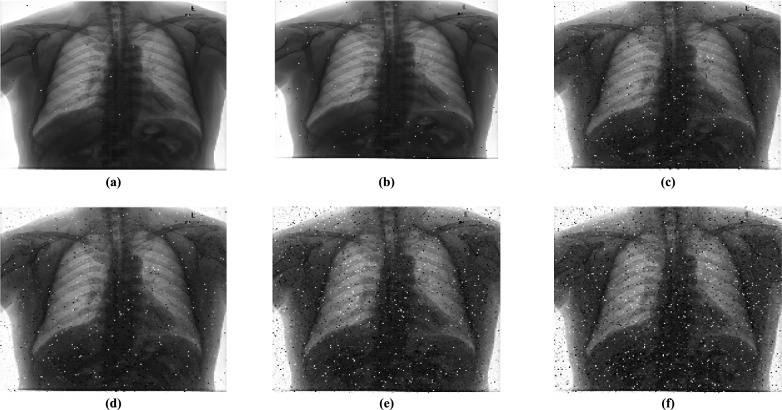
Decrypted image of Salt and pepper noise at noise intensity of (**a**)1% (**b**) 3% (**c**) 10%; Decrypted image of speckle noise at noise intensity of (**d**)1%, (**e**) 3%, (**f**) 10%.

Analyzing noise attacks^45^ is essential for evaluating the reliability of encryption algorithms, especially in real-world communication scenarios where various types of noise attack encrypted images. This proposed algorithm examines the effects of speckle and salt-and-pepper noise on encrypted images. Salt-and-pepper noise visibly distorts images by randomly replacing pixel values with extreme values, either black or white, with noise intensity of 1% 3% 10%**.** In contrast, speckle noise is a multiplicative noise that can severely degrade image quality, particularly at higher noise levels. This type of noise is introduced with strengths of 1% 3% and 10%**.**, causing granular changes in pixel values and significantly reducing the quality of the decrypted image. Despite noise attacks, most of the source image remains in the decrypted image. Analyzing the effects of these types of noise on the decrypted image allows us to assess the encryption algorithm’s effectiveness and resilience in protecting image authenticity under challenging conditions, as shown in Fig. 16**.**

##### Geometric attack analysis

The three types of geometric attacks on the cipher images also evaluate our proposed method’s security. Each distorted cipher image generated by a geometric attack was created as follows as:

- Rotation by ± 10°
- Translation by ± 10 pixels along both axes
- Scaling by factors of 0.8x, 0.9x

**Fig. 17:**
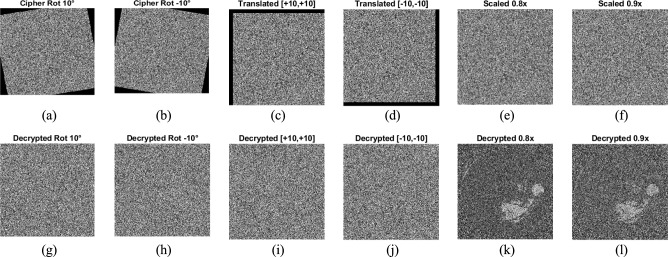
(**a**) &(**b**) Cipher image rotated by ± 10° (**c**) & (**d**) Translated cipher image by ± 10 in both axes (**e**)&(**f**) Cipher image scaled by 0.8x,0.9x (**g**) & (**h**) Decrypted image of (**a**&**b**), (**i**) &(**h**) Decrypted image of (**c** &**d**), (**k**)&(**l**) Decrypted image of (**e** &**f**).

The geometrically attacked cipher image was decrypted with the correct key. Figure 17. shows the attacked cipher (top row) and the decrypted result (bottom row). In each case, the decrypted image is the same as the noise-like encrypted image, demonstrating that any geometric deviation restricts the exact decryption of the image. The parameter evaluation is performed for the random decrypted images, which give NPCR > 99.6% and UACI > 33%, quantitatively confirming the avalanche effect under strong geometric disruption. The results ensure that the proposed encryption method effectively withstands rotation, translation, and scaling attacks.

### Security analysis

#### Key sensitivity analysis

**Fig. 18:**
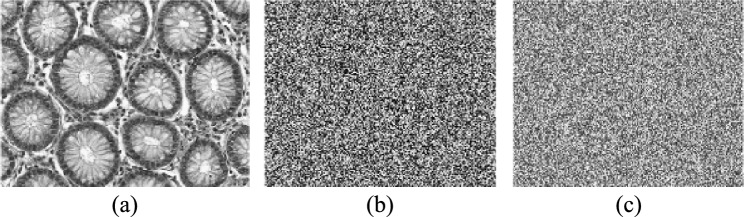
(**a**) Original image (**b**) Decrypted image with change in key x, and (**c**) Decrypted image with a change in key y.

The secure image encryption systems must have a key sensitivity factor, guaranteeing that even the slightest alteration to the encryption key produces an entirely different result. It ensures that an incorrect key cannot adequately decrypt the image, offering a strong defence against unwanted access. The experiment demonstrated the encryption technique’s extraordinary sensitivity to key variations when changing the keys x and y during decryption, which failed to generate the original image shown in Fig. 18. The algorithm demonstrates how much the system depends on exact key values to protect against brute-force attacks and maintain data integrity. This sensitivity is necessary to maintain the complexity and unpredictability of existing cryptographic systems.

The Fig. 17 demonstrates the effect of key variation by using the wrong key x and key y with a small change of **10**^**–6**^ in the algorithm, which results in the incorrect prediction of the original image and validates that the algorithm is highly sensitive to the wrong key value.

**Table:** 

Correct key	Wrong Key 1 (x altered)	Wrong Key 2 (y altered)
x = − 1.001050000000000, y = − 1.039000000000000, z = 0.990050000000000, u = − 1.019500000000000, v = − 1.026550000000000	**x = − 1.001051000000000,** y = − 1.039000000000000, z = 0.990050000000000, u = − 1.019500000000000, v = − 1.026550000000000	x = − 1.001050000000000, **y = − 1.039001000000000**, z = 0.990050000000000, u = − 1.019500000000000, v = − 1.026550000000000x

#### Key space analysis

The attacker has access to all encryption systems except the encryption key, which is crucial from the security perspective to protect the key. The brute force attack^49^ is resisted if the key space is more than 2^128^.The proposed algorithm has five initial conditions (xo, yo, zo, uo, vo***)*** is specified to a precision of 10^15^, yielding 10^15^ ≈ 2^50^ possible values per parameter. Consequently, the combined real‐value key space is (10^15^)^5^ = 10^75^≈2^249^. Including the permutation, dynamic DNA rule and DNA flip further enlarges the space to beyond 2^500^. This key size far exceeds the 2^128^ benchmark, ensuring immunity to brute‐force attacks. Table.9 compares the key space for different encryption methods with our method. It shows that the proposed method enlarges the key space sufficiently to avoid brute-force attacks.

**Table 9:** Key Space Analysis.

References	Dimension	Key space
REF^7^.	720 × 288	2^256^
REF^10^.	512 × 512	2^186^
REF^11^.	256 × 256	2^306^
REF^18^.	256 × 256	2^280^
REF^20^.	512 × 512	2^300^
REF^22^.	256 × 256	2^100^
REF^25^.	256X256	2^256^
REF^42^.	256X256	2^288^
**Proposed**	**256 × 256**	**2**^**500**^

### Quantitative analysis

#### Entropy

Entropy^50^ quantifies the randomness or unpredictability of pixel values, reflecting an image’s information content. Complex or textured regions exhibit higher entropy due to more significant variability, while smooth areas show lower entropy for the original image. Entropy measures information density by calculating the frequency intensity values in grayscale images. Comparing the entropy of original and encrypted images reveals encryption effectiveness; higher entropy in the encrypted image indicates successful data randomization and enhanced security. Table 10. presents the original medical test image entropy values and encrypted versions.

**Table 10:** Entropy, MSE, PSNR, NPCR and UACI of medical test encrypted images.

Data set name	Entropy		MSE	PSNR	NPCR	UACI
Test image	Test image	Encrypted image	Encrypted image	Encrypted image	Encrypted image	Encrypted image
Rite_test_01	**5.9992**	**7.9971**	**10,438.0255**	**7.9446**	**99.62**	**33.4793**
Rite_test_02	**5.4795**	**7.9969**	**10,180.6697**	**8.0530**	**99.60**	**33.5041**
Rite_test_03	**5.6988**	**7.9975**	**10,262.8907**	**8.0181**	**99.57**	**33.4746**
Rite_test_04	**5.6353**	**7.9970**	**10,107.4032**	**8.0844**	**99.60**	**33.4774**
Rite_test_05	**5.9840**	**7.9971**	**11,199.9969**	**7.6386**	**99.59**	**33.3316**
Rite_test_06	**5.5726**	**7.9969**	**10,204.8208**	**8.0427**	**99.61**	**33.3703**
Rite_test_07	**5.5003**	**7.9968**	**10,336.3203**	**7.9871**	**99.65**	**33.5477**
Rite_test_08	**5.6604**	**7.9972**	**10,249.8359**	**8.0236**	**99.58**	**33.5214**
Rite_test_09	**5.3715**	**7.9970**	**11,094.9197**	**7.6796**	**99.63**	**33.6601**
Rite_test_10	**5.4132**	**7.9973**	**10,324.7372**	**7.9920**	**99.60**	**33.511**
Drive_test_01	**5.5015**	**7.9975**	**10,211.0283**	**8.0401**	**99.61**	**33.5186**
Drive_test_02	**5.6253**	**7.9978**	**10,404.9462**	**7.9584**	**99.62**	**33.4219**
Drive_test_03	**5.2163**	**7.9976**	**11,036.3643**	**7.7025**	**99.61**	**33.434**
Drive_test_04	**6.0486**	**7.9980**	**10,490.6616**	**7.9228**	**99.57**	**33.4006**
Drive_test_05	**5.3943**	**7.9979**	**10,398.0024**	**7.9613**	**99.62**	**33.4225**
Drive_test_06	**5.5015**	**7.9975**	**10,321.8794**	**7.9932**	**99.59**	**33.4187**
Drive_test_07	**5.6253**	**7.9978**	**10,443.6278**	**7.9423**	**99.62**	**33.4947**
Drive_test_08	**5.6322**	**7.9975**	**10,317.0931**	**7.9952**	**99.65**	**33.3956**
Drive_test_09	**5.4132**	**7.9978**	**10,257.7693**	**8.0203**	**99.61**	**33.482**
Drive_test_10	**5.5975**	**7.9979**	**10,851.6779**	**7.7758**	**99.61**	**33.4673**
Drive_test_11	**6.0019**	**7.9976**	**10,387.2889**	**7.9658**	**99.60**	**33.3911**
Drive_test_12	**5.4805**	**7.9978**	**10,219.1024**	**8.0367**	**99.63**	**33.3348**
Drive_test_13	**5.7026**	**7.9979**	**10,229.2040**	**8.0324**	**99.57**	**33.427**
Drive_test_14	**5.6361**	**7.9974**	**10,178.5687**	**8.0539**	**99.65**	**33.6188**
Drive_test_15	**5.9901**	**7.9980**	**11,309.3349**	**7.5964**	**99.59**	**33.5505**
Drive_test_16	**5.5763**	**7.9977**	**10,220.0901**	**8.0363**	**99.59**	**33.3081**
Drive_test_17	**5.4982**	**7.9975**	**10,344.1948**	**7.9838**	**99.59**	**33.3338**
Drive_test_18	**5.6616**	**7.9971**	**10,375.3818**	**7.9708**	**99.62**	**33.5552**
Drive_test_19	**5.3768**	**7.9978**	**11,128.5554**	**7.6664**	**99.62**	**33.4723**
Drive_test_20	**5.4164**	**7.9976**	**10,347.0162**	**7.9827**	**99.58**	**33.4855**
Breast Image	**7.5544**	**7.9985**	**10,377.9539**	**7.9697**	**99.60**	**33.5208**
Gland Image	**6.2898**	**7.9992**	**13,168.4999**	**6.9354**	**99.63**	**33.4209**
Chest Image	**7.1983**	**8.0000**	**11,079.5463**	**7.6856**	**99.61**	**33.4555**
Brain Image	**3.2372**	**7.9920**	**17,751.0892**	**5.6386**	**99.61**	**33.3281**

23 $$\text{H }= -\sum_{k=1}^{N}{P}_{k }{log}_{2} {(p}_{k})$$

### Mean squared error and peak signal noise to ratio

To quantify their distortion, the Mean Squared Error (MSE) computes the average squared difference between the original and encrypted images’ respective pixel values. It offers an accurate estimate of image quality degradation by providing a clear metric for assessing how much the encrypted image differs from its original counterpart.

24 $$MSE= \frac{1}{R*C}\sum_{i=1}^{R}\sum_{j=1}^{C}{({I}_{1}\left(i,j\right)-{K}_{1}\left(i,j\right))}^{2}$$

where ${{\varvec{I}}}_{1}\left({\varvec{i}},{\varvec{j}}\right)$ and ${{\varvec{K}}}_{1}\left({\varvec{i}},{\varvec{j}}\right)$ are the pixel values at the position $\left({\varvec{i}},{\varvec{j}}\right)$ R and C represent the image dimensions in the original and encrypted images. It is difficult for unauthorized parties to recover or identify any portion of the original content when the MSE value is high, indicating that the encrypted image differs greatly from the original. Based on the MSE, PSNR^51^ offers a logarithmic assessment of the encrypted image’s quality compared to the original.

### Peak signal noise to ratio

PSNR, which is commonly measured in decibels (dB), is used to evaluate the degree of distortion in the encryption^52^**.**

25 $$PSNR=10{log}_{2}\left(\frac{{MAX}^{2}}{MSE}\right)$$

where MAX represents the maximum possible pixel value (255 for an 8-bit image), a larger degree of distortion from the original image is indicated by a lower PSNR, which implies an efficient encryption procedure. Table 10. shows the MSE and PSNR values of various medical images. For secure encryption, a high MSE and low PSNR value work together to ensure that the encrypted image cannot be identified from the original.

### Differential attack analysis

A differential attack is a cryptanalysis technique that exploits the relationship between the original and encrypted images by introducing small changes to the original image and analyzing their impact on the encrypted output^53^**.** Secure encryption technology ensures that even minor changes to the source image produce significant and unpredictable alterations in the encrypted image. Metrics such as the Number-of-Pixels Change Rate (NPCR) and Unified Average Changing Intensity (UACI) measure the effectiveness of an encryption method in combating such attacks.

#### NPCR

When a single pixel value in the source image changes, NPCR calculates^54^ the proportion of pixels that change in the encrypted image. This test measures the avalanche effect, or how sensitive the encryption process is for slight modification of the original image pixel.

26 $$NPCR = \sum_{i,j}\frac{{D}_{1}\left(i,j\right)}{R*C}$$

27 $${D}_{1}\left(i,j\right)=\left\{\begin{array}{c} 0, if {c}_{1} (i,j )= {c}_{2}(i,j) \\ 1, if {c}_{1}\left(i,j\right) \ne {c}_{2}(i,j)\end{array}\right.$$

where R and C are the image dimensions (height and width),

${\text{D}}_{1}\left(\text{i},\text{j}\right)$ is the change at each pixel, as defined as the total number of pixel variations between the two encrypted images counted, and the percentage result is displayed.

#### UACI

Unified Average Changing Intensity measures^50^ the mean pixel value difference between two encrypted images created from original images with a slight difference (such as a single pixel alteration). UACI measures the intensity of pixel changes, whereas NPCR counts them.

28 $$\text{UACI }= \sum_{i,j}\frac{\left|{c}_{1} \left(i,j \right)-{c}_{2}(i,j)\right|}{255 X R*C}\times 100\text{\%}$$

where ${\text{c}}_{1} \left(\text{i},\text{j}\right)$ and ${\text{c}}_{2}(\text{i},\text{j})$ are the image pixel values of the two encrypted images, R and C represent the image dimensions. The NPCR and UACI values of different medical images are given in Table 10.

The results in Table 10 show that NPCR values vary from 99.57% to 99.65%, revealing that all the pixel values between two different ciphered images obtained from the single bit change in the original image, resulting in different values for the encrypted image satisfy strong sensitivity to the input changes. The UACI values vary from 33.31% to 33.66%. The low value of UACI shows that the algorithm results in moderate variations in the pixel brightness, which is very important in the security measures of image encryption. In the paper^42,55^, the theoretical ideal value of NPCR is 99.6094%, and the UACI value is approximately 33.46% for 8-bit greyscale images. The values obtained by our proposed method for the various medical images equal the ideal value of the reference paper, proving the robustness of our process in defending against differential attack.

## Performance comparison with existing algorithms

Critical metrics like correlation coefficients, entropy, NPCR, and UACI are used to assess the suggested encryption algorithm on six different kinds of medical images (RITE, DRIVE, Breast, Gland, Chest, and Brain) and compare it with previous techniques are shown in Table 11**.** The proposed method matches and consistently surpasses current algorithms’ performance, obtaining uniformly low horizontal, vertical, and diagonal correlation values. The Brain image, in particular, suggests excellent encryption quality and minimal inter-pixel dependency, further reinforcing the superiority of our proposed algorithm. Entropy values obtained by the suggested approach are around the optimal value of 8, indicating good unpredictability and robustness against statistical attacks that should validate the proposed work. The Chest image, in particular, outperformed most current techniques, achieving an entropy score of 8.0000. The NPCR values, which measure resistance to differential attacks, consistently meet or exceed the crucial threshold of 99.6%, with the method recording values of about 99.61% for most images. The UACI value of the breast image is approximately 33.5208, which is within the critical range (~ 33.46%), and it shows the encryption method adaptability.

**Table 11:** Performance comparison.

	Method	Horizontal correlation	Vertical correlation	Diagonal correlation	Entropy	NPCR	UACI
Existing Algorithm	Ref^14^.	−0.0073	−0.0005	−0.002	7.9982	99.62	33.49
	Ref^15^.	−0.0009	−0.0009	−0.0013	7.9996	99.69	33.53
	Ref^16^.	−0.00098	−0.00042	- 0.00020	7.9991	99.60	50.0430
	Ref^17^.	− 0.00148	0.0038	− 0.00019	7.995	99.47	49.90
	Ref^19^.	−0.0065	0.0026	−0.0297	7.9992	99.59	33.45
	Ref^41^.	0.0050	0.0090	0.0060	7.9993	99.51	25.2060
	Ref^42^.	0.0006	0.0006	0.0004	7.9989	99.60	33.5003
	Ref^43^.	0.0014	0.0018	0.0016	7.9989	99.61	33.5003
	Ref^44^.	−0.0007	0.0013	−0.0085	7.9979	99.61	33.4364
	Ref^56^.	0.0009	0.0014	0.0004	7.9992	99.68	33.5539
	Ref^57^.	0.0008	0.0046	0.0032	7.9976	99.66128	33.55964
Proposed Algorithm for different medical images	RITE Dataset	−0.0018	−0.0002	0.0007	7.9971	99.61	33.4878
	DRIVE Dataset	−0.0007	0.0006	0.0004	7.9977	99.61	33.4467
	Breast Image	−0.0020	−0.0003	−0.0018	7.9985	99.60	33.5208
	Gland Image	−0.0008	0.0008	−0.0027	7.9992	99.63	33.4209
	Chest Image	−0.0001	0.0006	−0.0002	8.0000	99.61	33.4555
	Brain Image	−0.0049	−0.0044	−0.0111	7.9920	99.61	33.3281

*The average values for Rite dataset and Drive dataset.

The proposed work is strong and successful in securing medical data, which results in pixel decorrelation, significant uncertainty and resistance to differential attacks. The research performs better than conventional methods, ensuring robustness and optimal encryption for sensitive medical images.

### Computational complexity and time performance analysis

An algorithm’s computation time^58^ is analyzed to assess its efficiency. In this proposed work, Critical region segmentation using U-Net and the encryption technique are the two phases of operations used to analyze computational overhead.

Critical Region Extraction (Segmentation Phase).

The computation time for the U-shaped encoder and decoder operation used to extract the critical regions is based on the depth and size of the input image.

29 $$Computational{\mkern 1mu} Complexity{\mkern 1mu} of{\mkern 1mu} segmentation{\mkern 1mu} = O{\mkern 1mu} \left( {L{\mkern 1mu} {\mkern 1mu} N} \right)$$

L—Number of convolution layers in the customized U-Net model. N x N -represents the shape of the medical image. The segmentation precision and computation overload are balanced by a medical image size of 256 × 256.

Encryption Phase (5D Hyperchaotic and DNA Encoding):

After key generation using segmentation, encryption is performed using hyper chaotic systems and DNA-based operations. This process involves generating chaotic sequences, scrambling pixel positions, performing DNA encoding, dynamic DNA flipping, and dynamic XOR-based diffusion.

30 $$Computational \, Complexity \, of \, Encryption \, = O \, \left( {P \, log \, P} \right)$$

where the number of pixels in the input image is given as P, among the various encryption steps, the chaotic pixel sorting operation has the most significant impact on complexity, accounting for the logarithmic term.

The total computational complexity of the proposed algorithm is given in Eq. (31)

31 $$Total\;Computational \, Complexity \, = O \, \left( {L \times N^{2} + P \, log \, P} \right)$$

### Time complexity (execution time)

The platform used to evaluate the overall run time of the encryption algorithm:

- Segmentation phase for key generation: Google Colab with an NVIDIA Tesla T4 GPU
- Encryption phase: MATLAB R2021a on a Dell i5 CPU (4 cores, 16 GB RAM).

The computational infrastructure, which includes processor design, system software, memory footprint, implementation specifics, and build parameters, significantly impacts cipher performance measures like encryption latency and bandwidth. As a result, comparing ciphers across different hardware and software stacks produces inaccurate comparisons^56^. The execution time for a single 256 × 256 grayscale medical image is given in Table 12.

**Table 12:** Time complexity.

Process	Platform	Execution time
U-Net Segmentation	**Google Colab (Tesla T4)**	**0.03 s**
Encryption	**Dell i5 CPU (MATLAB)**	**2.9 s**
Total Time	**Cross-platform**	**2.93 s**

Although U-Net inference for a single 256 × 256 image requires ≈0.03 s on a mid-range GPU, segmentation is performed only once offline to derive the chaotic-map key. For real-time or large-scale deployments, segmentation can be accelerated via (1) model compression (structure simplification, quantization), (2) lightweight U-Net variants (depth wise separable convolutions), (3) tiled or batch inference, and (4) deployment on hardware accelerators (GPUs, FPGAs). These strategies yield 2–10 × increase in speed with minimal accuracy trade-offs.

### Feasibility on embedded medical IoT platforms:

The suggested encryption method is resource-efficient and customizable, facilitating deployment for embedded computing systems even though our work has been tested on general computing hardware. After architecture-aware refinement, devices like the NVIDIA Jetson Nano (with GPU acceleration and 4 GB RAM) and Raspberry Pi 4 (with a quad-core ARM Cortex-A72 processor and 2–8 GB RAM) may be able to support the algorithm. Future research will need to address actual deployment, but we predict that hardware acceleration, fixed-point implementation, and practical coding techniques may allow real-time processing on such devices, making the proposed algorithm feasible for safe medical IoT systems.

## Conclusion and future scope

This paper shows that the proposed medical image encryption algorithm combines a new 5D hyperchaotic system with a U-Net network, demonstrating excellent security and computational efficiency. The confidential image extracted from the medical image is used as the chaotic system’s initial condition to prevent the medical image from being accessed by unauthorized people. The encrypted image’s high entropy, which is close to 8, indicates that it is more unpredictable than the original image. Additionally, Correlation coefficients close to zero in all directions indicate that the cypher image has minimal pixel correlation, disrupting patterns and enhancing security, and correlation analysis demonstrates a uniform distribution. A desirable aspect of encryption is the notable distortion between the original and encrypted images, characterized by low PSNR values and high MSE. The proposed algorithm achieves an NPCR of 99.67% and a UACI of 33.99%, which shows that it is sensitive to pixel-level changes to prevent differential assaults. These values are close to the critical threshold. The algorithm generates a larger key space of 2^500^ which is far beyond the 2^128^ to block the brute force attack. The algorithm (U-Net segmentation + encryption) completes approximately 2.93 s for 256 × 256 images, validating its suitability in real-time medical applications. Furthermore, the total computational complexity O (L × N^2^ + P log P) ensures that the algorithm is scalable for real-time implementation. A performance comparison of encryption algorithms with existing techniques confirms their security and effectiveness in safeguarding confidential medical images in real-world applications.

The proposed research is limited only to 2D images. Future research will use 3D U-Net and volumetric chaotic integrating to expand this method to 3D medical data (such as MRIs and CT scans), guaranteeing safe encryption of depth-wise structures. Lightweight segmentation and quick chaos-based operations will also be investigated for real-time encryption of video streams (such as ultrasound). The current method will improve in the future by using several dynamic strategies for dispersion and rearrangement procedures to achieve better results. Further investigations might use advanced deep learning models to generate complex, image-specific encryption keys obtained from anatomical data to increase medical image encryption’s versatility and safety. In future work, we plan to integrate the post quantum cryptographic techniques with our hybrid chaotic system to ensure resilience against the quantum attacks. Integrating real-time encryption methods for telemedicine and cloud storage paves the way for future innovations in patient data management to reinforce security and performance without ensuring significant advances in continuing research.

## Data Availability

The datasets generated during and/or analyzed during the current study are available from the corresponding author upon reasonable request. 2. All data generated or analyzed during this study are included in this published article. The datasets analyzed during the current study are available in https://www.sciencedirect.com/science/article/pii/S0933365721001093?via%3Dihub; https://link.springer.com/chapter/10.1007/978-3-642-40763-5_54https://drive.grand-challenge.org/https://www.sciencedirect.com/science/article/pii/S2352340919312181https://ieeexplore.ieee.org/document/7,109,172https://ieeexplore.ieee.org/document/6,616,679.
